# Comparative Genome Analysis of Three *Halobacillus* Strains Isolated From Saline Environments Reveal Potential Salt Tolerance and Algicidal Mechanisms

**DOI:** 10.1111/1758-2229.70121

**Published:** 2025-06-15

**Authors:** Saru Gurung, Chang‐Muk Lee, Hang‐Yeon Weon, So‐Ra Han, Tae‐Jin Oh

**Affiliations:** ^1^ Department of Life Science and Biochemical Engineering SunMoon University Asan Republic of Korea; ^2^ Bio Big Data‐Based Chungnam Smart Clean Research Leader Training Program SunMoon University Asan Republic of Korea; ^3^ Agricultural Microbiology Division, National Institute of Agricultural Sciences Rural Development Administration Jeonju Republic of Korea; ^4^ Technology Services Division, National Institute of Agricultural Sciences Rural Development Administration Jeonju Republic of Korea; ^5^ Genome‐Based BioIT Convergence Institute Asan Republic of Korea; ^6^ Department of Pharmaceutical Engineering and Biotechnology SunMoon University Asan Republic of Korea

**Keywords:** algicidal activity, comparative genome analysis, *Halobacillus* sp., halotolerance, stress response

## Abstract

Harmful algal blooms (HABs) pose a significant global threat to water ecosystems, prompting extensive research into their inhibition and control strategies. This study presents genomic and bioinformatic analyses to investigate the algicidal potential and elucidate the survival mechanisms in harsh conditions of newly identified *Halobacillus* species three strains (SSTM10‐2^T^, SSBR10‐3^T^, and SSHM10‐5^T^) isolated from saline environments. Moreover, genomic and bioinformatic analyses were conducted to elucidate their survival mechanisms in harsh conditions. Moreover, comparative genomic analysis revealed a diverse set of orthologous genes, with a core genome primarily associated with metabolism and information processing. Pangenome analysis highlighted accessory and unique genes potentially involved in environmental adaptation and stress response. Functional annotation using KEGG pathways identified genes linked to xenobiotic compound degradation, stress tolerance, and salt adaptation. Additionally, the study elucidated potential mechanisms underlying algicidal activity, implicating Carbohydrate‐Active enZYmes (CAZymes), cytochrome P450 oxidases (CYP), and quorum sensing (QS) systems. Finally, analysis of KEGG pathways related to microcystin degradation suggested the strains' capacity to mitigate HABs. Thus, this research enhances understanding of the genomic diversity, phylogeny, and functional characteristics of *Halobacillus* species, offering insights into their ecological roles and potential applications in biotechnology and environmental management.

## Graphical abstract

Following the previous complete genome analysis, this research provides the function of new *Halobacillus* species. Three strains were identified: *Halobacillus shinanisalinarum* sp. nov. SSTM10‐2^T^, *Halobacillus salinarum* sp. nov. SSBR10‐3^T^, and *Halobacillus amylolyticus* sp. nov. SSHM10‐5^T^. Highlighting comparative genomic and pangenome analysis of *Halobacillus* species, genes involved in xenobiotic compound degradation, focusing on algicidal properties, were identified.

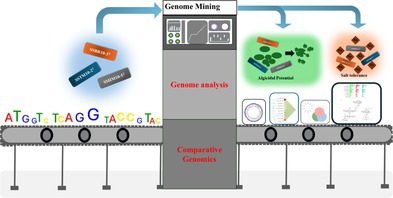

## 1 Introduction

*Halobacillus* was first described in 1996 by Spring et al. *Halobacillus* is a genus of gram‐positive, spore‐forming rod‐shaped bacteria presently comprising 82 species (accessed on April 3, 2025), as documented on the List of Prokaryotic Names with Standing in Nomenclature (LPSN) website (https://www.bacterio.net) (Parte et al. 2020). They are distinctly set apart from organisms in closely related genera due to their predominant menaquinone being MK‐7 as well as the presence of anteiso‐C15:0, iso‐C15:0, C16:0, and iso‐C16:0 as primary fatty acids in their cell wall (An et al. 2007; Yoon et al. 2008, 2007). Halophiles such as haloarchaea and halophilic bacteria (Chen et al. 2009) are primarily found in hypersaline and marine environments rich in sodium chloride (NaCl) (Edbeib et al. 2016), including salt/saline lakes (Edbeib et al. 2016), mangrove ecosystems (Khan et al. 2009), saline springs (Díaz‐Cárdenas et al. 2010), topan salt (Woo et al. 2017), and more. These extremophiles thrive in high‐salt environments by adopting two primary osmoregulatory strategies to withstand osmotic stresses (Corral et al. 2020; Siglioccolo et al. 2011). The first strategy involves the “salt‐in” approach, characterized by accumulating inorganic ions, notably K^+^ and Cl^−^, within the cell (Brown 1976; Gunde‐Cimerman et al. 2018; Lanyi 1974). The second strategy centres on accumulating compatible solutes such as sugars (trehalose and polyols) and α‐ and β‐amino acids (glutamine, glutamate, proline, and derivatives like betaine and ectoine) (Gunde‐Cimerman et al. 2018; Roberts 2005; Weinisch et al. 2018).

Most research on halophilic bacteria, including *Halobacillus*, has focused on their potential for bioremediating xenobiotics in highly saline environments (Li et al. 2012; Moreno et al. 2012). Researchers have demonstrated the catabolic versatility of these bacteria in degrading aromatic compounds, further indicating their potential for xenobiotic degradation (García et al. 2005; Shukla and Singh 2020). Recent metagenomic studies, such as Sierra et al. (2022), have revealed pigment‐rich polyextremophiles with wide‐ranging metabolic adaptations, while Shen et al. (2022) have provided functional insight into *Halobacillus trueperi* S6, highlighting its potential in biotechnological applications. However, remarkably, no published reports have yet explored the genomic potential of the *Halobacillus* genus for combating algae, highlighting a critical gap in current research.

In general, eutrophication of water bodies often leads to the proliferation of HABs, commonly known as red tide events, primarily induced by toxic algal species. These occurrences are prevalent on a global scale. The increasing occurrence of large‐scale HABs has significant ecological consequences, including a substantial reduction in underwater light penetration, which can damage seagrass beds (Heisler et al. 2008). Additionally, these algal blooms release considerable amounts of toxic substances into the aquatic environment, leading to mass mortality events among marine organisms (Karlson et al. 2021). Thus, the ecological repercussions (Kazmi et al. 2022) and adverse economic impacts of HABs have garnered widespread attention recently (Hoagland and Scatasta 2006; Song et al. 2021). Previously, chemical and physical methods were applied to control HABs. However, the need for biological control arose due to their cost‐effectiveness and as a potential solution involving utilizing specific bacterial strains known as algicidal bacteria (Coyne et al. 2022; Song et al. 2023). For example, Li et al. (2015) isolated *Bacillus* sp. strain Lzh‐5 and demonstrated that compounds from this strain exhibited strong algicidal activity against *Microcystis aeruginosa*. Also, *Enterobacter cloacae* NP23 (Liao et al. 2015a) and *Gibberella moniliformis* AN11 (Liao et al. 2015b) had high specificity to inhibition of *Chlorella pyrenoidosa* by disrupting host oxidative balance (i.e., inhibition of antioxidase activities). Thus, some bacteria have already been proven effective, and further investigation and in‐depth research on bacteria with algicidal potential are needed.

Therefore, our research aims to fill this gap by analysing the genomes of three novel *Halobacillus* strains: *Halobacillus shinanisalinarum* sp. nov. SSTM10‐2^T^, *Halobacillus salinarum* sp. nov. SSBR10‐3^T^, and *Halobacillus amylolyticus* sp. nov. SSHM10‐5^T^. This paper is a follow‐up study presenting the comparative genomic analysis results of the three strains following the publication of their complete genomes, and we aim to hypothesize a possible mechanism through which these bacteria may exhibit algicidal activity across three specific strains.

## 2 Experimental Procedures

### 2.1 Bacterial Strains Isolation and Genome Sequencing Information

Our study builds on Kim et al.'s work, utilizing the same strains for further comparative genomic analysis (Kim et al. 2023). The bacterial strains SSBR10‐3^T^, SSTM10‐2^T^, and SSHM10‐5^T^ have been identified as novel species and proposed with names *Halobacillus shinanisalinarum* sp. nov. (SSTM10‐2^T^ = DSM 114354^T^ = KACC 21936^T^ = NBRC 115505^T^), *Halobacillus salinarum* sp. nov. (SSBR10‐3^T^ = DSM 114353^T^ = KACC 21935^T^ = NBRC 115504^T^), and *Halobacillus amylolyticus* sp. nov. (SSHM10‐5^T^ = DSM 114355^T^ = KACC 21937^T^ = NBRC 115506^T^). The comprehensive genomic sequences, annotated using the NCBI Prokaryotic Genome Annotation Pipeline, are available in the NCBI GenBank database under accession numbers CP095073, CP095074, and CP095075, respectively.

### 2.2 Genome Annotation and Comparative Genome Analysis

For the comparative genomic analysis, the complete genomes of 10 *Halobacillus* species (including nucleotide and protein sequences) were downloaded from NCBI, as they were available at the time of our study. Moreover, the pan‐genome analysis was performed by employing the BPGA software package version 1.3 (Chaudhari et al. 2016) on all the complete genomes of *Halobacillus* species, utilizing the default parameters with an identity cutoff value of 0.5 for clustering. Functional KEGG analysis and its distribution in the core, pan, and accessory genome were performed using the advanced option in the BPGA software package. Subsequently, pathway analysis was conducted using KEGG.

### 2.3 Genome Annotation and Functional Prediction

Diverse annotation tools were utilized to investigate the genomic features of the three strains SSBR10‐3^T^, SSTM10‐2^T^, and SSHM10‐5^T^. First, a circular genome map was obtained using the CGview server in Proksee (https://proksee.ca/) (Stothard and Wishart 2005). Additionally, the Rapid Annotation using Subsystem Technology (RAST) server (Aziz et al. 2008), the Cluster of Orthologous Groups of proteins (COG) from the eggnog 5.0 database (Huerta‐Cepas et al. 2019), the Kyoto Encyclopedia of Genes and Genomes (KEGG) (Kanehisa and Goto 2000) with a cutoff value of 0.01, and the National Center for Biotechnology Information (NCBI) were used for functional annotation of genes and proteins. The Basic Local Alignment Search Tool (BLAST) and non‐redundant protein sequence (nr) database (McGinnis and Madden 2004) were used for functional gene analysis. The dbCAN3 server (Zheng et al. 2023) was used to conduct Carbohydrate‐Active enZYme (CAZyme) analysis, selecting two tools, HMMER: dbCAN (E‐Value < 1e‐15, coverage > 0.35) and DIAMOND: CAZy (E‐Value < 1e‐102). The OrthoVenn3 web server (Sun et al. 2023) was utilized for pangenome analysis, gene ontology of all predicted protein‐coding genes, and unique gene search across strains SSTM10‐2^T^, SSBR10‐3^T^, and SSHM10‐5^T^. The tool uses the DIAMOND algorithm to perform all‐against‐all sequence comparison and OrthoMCL (Li et al. 2003) to identify orthologous genes based on comparison with default parameters (e‐value = 1e‐2, inflation value = 1.5, enabled annotation and protein similarity network and disabled cluster relationship). The secondary metabolite gene clusters were predicted using antiSMASH bacterial version 7.1.0 with default parameters (Blin et al. 2023). A metagenomic study focusing on bacterioplankton taxa and pathways involved in microcystin degradation in Lake Erie compared KEGG pathways microcosms with and without microcystin (Mou et al. 2013). The genomes of *Halobacillus* strains SSTM10‐2^T^, SSBR10‐3^T^, and SSHM10‐5^T^ were also examined for these pathways using the KEGG pathway database.

### 2.4 Phylogenetic Analysis

The phylogenetic relationships of *Halobacillus* strains SSTM10‐2^T^, SSBR10‐3^T^, and SSHM10‐5^T^ were investigated through 16S rRNA gene sequence analysis. The longest full‐length 16S rRNA gene sequence (≥ 1400 bp, without ambiguous bases) was selected for each genome. The sequences were initially aligned using the NCBI database's BLAST and further analysed via the EzBioCloud database (https://ezbiocloud.net) using the 16S‐based ID feature. Multiple sequence alignment was performed using MUSCLE (Edgar 2004), and a phylogenetic tree was constructed using the maximum likelihood tree with 1000 bootstrap replicates in MEGA X (Kumar et al. 2018). Comparative analysis with other *Halobacillus* species and others registered in NCBI confirmed the close relationship of the newly identified strains within the genus *Halobacillus*. To further validate the taxonomic position of the strains, whole‐genome sequences of closely related *Halobacillus* species were retrieved from NCBI (https://www.ncbi.nlm.nih.gov), and genome‐based comparisons were carried out. Average Nucleotide Identity (ANI) values were calculated using OrthoANI in Orthologous Average Nucleotide Identity Tool (OAT) (Lee et al. 2016), while digital DNA–DNA hybridization (dDDH) values were estimated using Genome‐to‐Genome Distance Calculator (GGDC) 3.0 from the Leibniz Institute DSMZ (https://ggdc.dsmz.de/ggdc_background.php) with default parameters (Auch et al. 2010).

## 3 Results

### 3.1 General Feature and Phylogenetic Analysis, Calculation of ANI and dDDH of *Halobacillus* sp. SSTM10‐2^T^ , *Halobacillus* sp. SSBR10‐3^T^ , and *Halobacillus* sp. SSHM10‐5^T^

**FIGURE 1:**
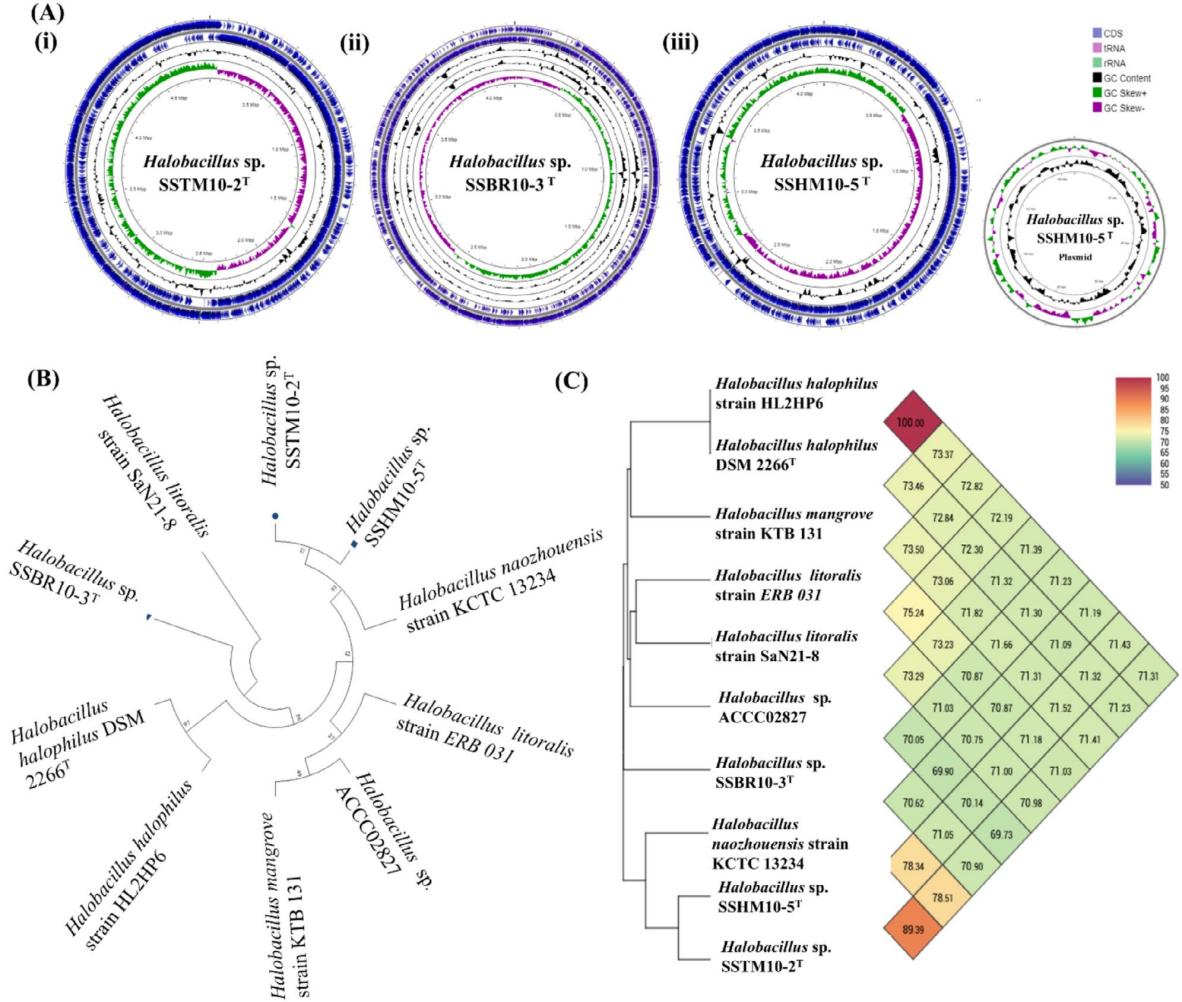
Circular map of (A) (i) *Halobacillus shinanisalinarum* SSTM10‐2^T^, whole chromosome; (ii) *Halobacillus salinarum* SSBR10‐3^T^, whole chromosome; and (iii, iv) *Halobacillus amylolyticus* SSHM10‐5^T^, whole chromosome and plasmid. (B) 16 s rRNA phylogenetic tree and (C) Orthologous Average Nucleotide Identity (ANI). Phylogenetic tree was generated using maximum likelihood method with cutoff value of 50%, constructed using MEGAX based on 16S rRNA sequences. ANI percentage was calculated using OrthoANI in OAT (Orthologous Average Nucleotide Identity Tool).

As the focus of this study, detailed information on features of the three strains *Halobacillus* sp. SSTM10‐2^T^, *Halobacillus* sp. SSBR10‐3^T^ and *Halobacillus* sp. SSHM10‐5^T^ is provided (Table 1). The complete genomes of both *Halobacillus* sp. SSTM10‐2^T^ and *Halobacillus* sp. SSBR10‐3^T^ consist of one circular chromosome, while *Halobacillus* sp. SSHM10‐5^T^ consists of one circular chromosome and one plasmid (Figure 1A (i–iii)). Further details on these strains can be found in a study conducted by Kim et al. (2023).

**TABLE 1:** General features and genomic assembly of *Halobacillus shinanisalinarum* SSTM10‐2^T^, *Halobacillus salinarum* SSBR10‐3^T^ and *Halobacillus amylolyticus* SSHM10‐5^T^.

Genome feature	*Halobacillus shinanisalinarum* SSTM10‐2^T^	*Halobacillus salinarum* SSBR10‐3^T^	*Halobacillus amylolyticus* SSHM10‐5^T^	
	Chromosome	Chromosome	Chromosome	Plasmid
Genome size (bp)	4,887,025	4,240,228	4,154,097	145,254
GC content (mol %)	40.50	42.50	40.50	37.15
Total number of genes	4892	4382	4340	144
Total number of proteins	4563	4133	4101	129
tRNA	69	66	70	1
rRNA	27	21	24	0
Scaffold N50 (Mb)	4.9	4.2	4.2	N/A
Scaffold L50	1	1	1	N/A
Total number of CDS	4776	4265	4244	143
Number of CAZyme genes	101	105	94	N/A
KEGG metabolic pathways	221	221	221	N/A
Accession number	CP095073	CP095074	CP095075	—

*Note:* The total number of genes, proteins, tRNAs, rRNAs, and coding sequences (CDSs) present in the plasmid of *Halobacillus amylolyticus* strain SSHM10‐5ᵀ were manually counted using the GenBank file retrieved from NCBI.

To date (October 10, 2024), 59 *Halobacillus* strains have been submitted to NCBI, comprising 10 with complete genomes, 34 with contig‐level assemblies, and 15 strains with scaffold‐level assemblies. The size of the analysed genomes ranged from 3.6 to 4.8 Mb. The general features of 10 strains in this study are presented (Supplementary Table S1). Three strains contained two plasmids, four had one plasmid, and the remaining three had none.

A maximum likelihood phylogenetic analysis based on the 16 s rRNA gene sequence was performed using MEGAX to identify the three strains, showing SSTM10‐2^T^ closely related to SSHM10‐5^T^ in a clade with *Halobacillus naozhouensis* strain KCTC13234. In comparison, SSBR10‐3^T^ formed a separate branch (Figure 1B). ANI values were compared between our strains and seven other *Halobacillus* strains with genomic data, revealing an ANI of 89.39% between SSM10‐2^T^ and SSBR10‐3^T^. In contrast, values with *Halobacillus naozhouensis* strain KCTC13234 were 78.51% for SSM10‐2^T^ and 78.34% for SSBR10‐3^T^, below the 96% delineation threshold. As for SSHM10‐5^T^, the highest ANI value with any other strain was 71.66%, similarly below the species threshold (Figure 1C). Additionally, dDDH values for *Halobacillus* sp. SSTM10‐2^T^, *Halobacillus* sp. SSBR10‐3^T^, and *Halobacillus* sp. SSHM10‐5^T^ ranged from 14% to 49.3% when compared to other genomes (Supplementary Table S2). Since a dDDH value above 70% typically indicates species identity with a reference strain, the low dDDH values further supported that these strains are distinct species. Specifically, the dDDH values were 49.3 for SSTM10‐2^T^ and SSHM10‐5^T^ and 14.7 for SSBR10‐3^T^.

### 3.2 Comparative Genomic Analysis of Orthologous Genes Among Three Strains of *Halobacillus* Species

**FIGURE 2:**
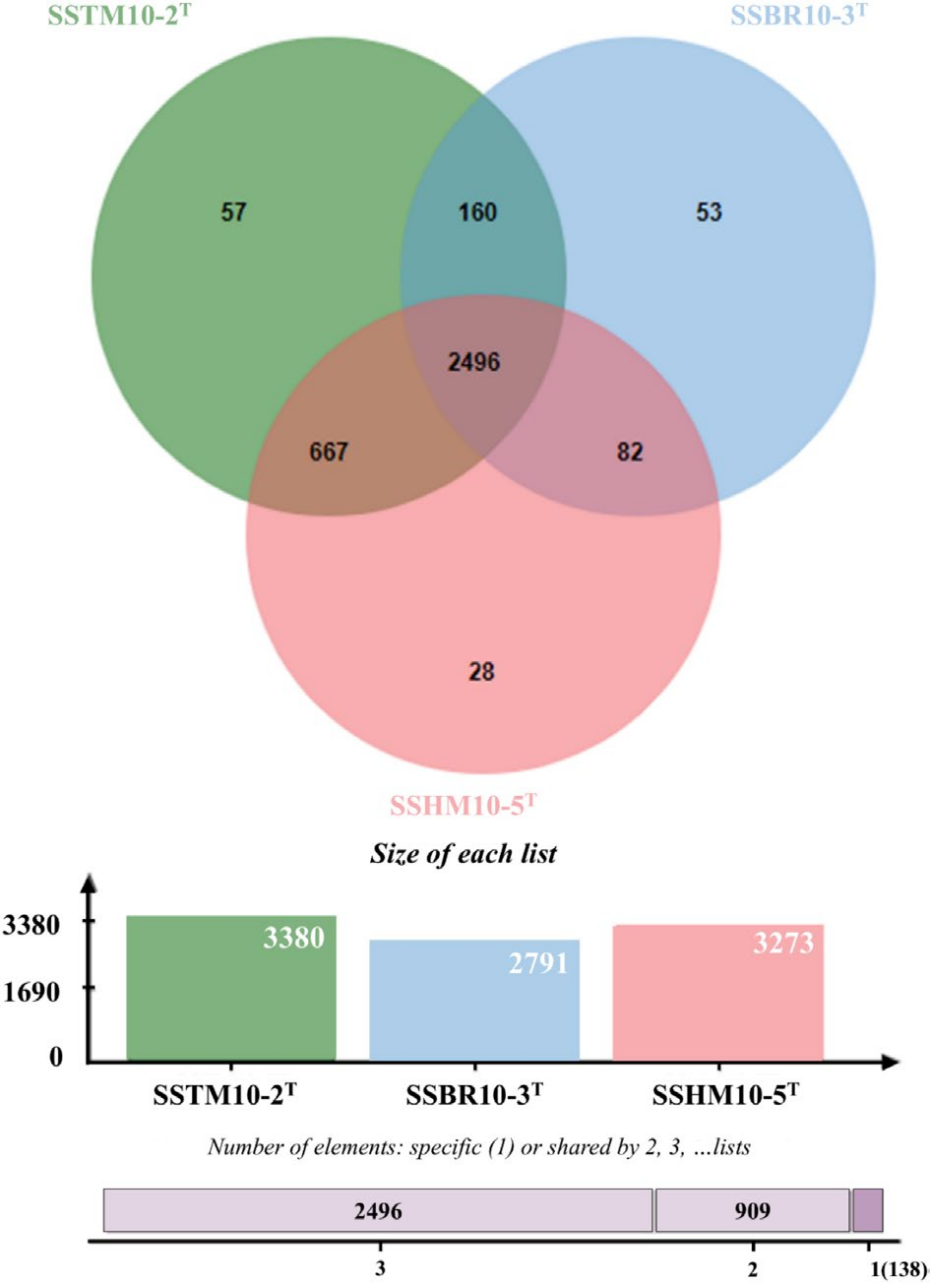
Venn diagram of *Halobacillus shinanisalinarum* SSTM10‐2^T^, *Halobacillus salinarum* SSBR10‐3^T^, and *Halobacillus amylolyticus* SSHM10‐5^T^. Venn diagram was constructed using orthoVenn3 with default settings.

Orthologous genes were analysed between three strains of *Halobacillus* species to elucidate genetic relationships and functional similarities among the strains. Protein sequences of the newly assembled *Halobacillus* strains SSTM10‐2^T^, SSBR10‐3^T^, and SSHM10‐5^T^ were used (Figure 2). The total number of orthologous gene clusters was 3380 for strain SSTM10‐2^T^, 2791 for strain SSBR10‐3^T^, and 3273 for strain SSTM10‐2^T^. The three *Halobacillus* species shared a total of 2496 clusters in common, while SSTM10‐2^T^ shared 2656 and 3245 gene clusters with SSBR10‐3^T^ and SSHM10‐5^T^, respectively, with an additional 57 specific gene clusters.

### 3.3 Pangenome Analysis of *Halobacillus* sp.

The pan‐genome analysis is a method that addresses genomic diversity by evaluating the core, accessory, and unique gene components within genomes. As postulated by Tettelin et al. (2005), the pan‐genome is conceptually defined as the comprehensive ensemble of genes encompassing the entirety of a microorganism's genomic repertoire. The core gene is frequently found in all strains, the accessory gene is present in two or more strains (Steinberg et al. 2022), and the unique gene is unique to individual species members (Armisén et al. 2008; Gonzales‐Siles et al. 2020). The pangenome of ten *Halobacillus* strains comprised 1572 core genes (6.24%) and 18,063 accessory genes (71.73%). The unique genome comprises 5542 genes (22.03%) (Table 2). Most strains showed low numbers of exclusively absent genes, ranging from 1 to 112. Core genes, which are highly conserved and prevalent across strains, are primarily involved in basic physiological functions, resulting in the overall prevailing phenotypes of the organisms (Basharat et al. 2018). On the other hand, accessory genes, while less prevalent, can confer specific phenotypes advantageous for bioremediation, such as encoding novel catabolic pathways promoting persistence in inhospitable environments (Kung et al. 2010). Unique genes, as the name suggests, are specific to individual strains. The strain SSTM10‐3^T^ harboured the highest number of unique genes (1172) compared to the other strains, followed by SSTM10‐2^T^ (790). Hence, gene exchange activities associated with potentially different approaches to adaptation and response to environmental conditions occur at the highest rate in this specific strain.

**TABLE 2:** Genes distribution of *Halobacillus* species calculated using BPGA‐pangenome pipeline.

S.N.	Organism name	No. of core genes	No. of accessory genes	No. of unique genes	No. of exclusively absent genes
1	*Halobacillus amylolyticus* SSHM10‐5^T^	1572	1787	490	30
2	*Halobacillus halophilus* DSM2266^T^	1572	2246	5	1
3	*Halobacillus halophilus* HL2HP6	1572	2241	13	2
4	*Halobacillus litoralis* ERB031	1572	1807	569	8
5	*Halobacillus litoralis* SaN21‐8	1572	1571	623	60
6	*Halobacillus mangrovi* KTB131	1572	1730	592	35
7	*Halobacillus naozhouensis* KCTC13234	1572	1841	590	17
8	*Halobacillus salinarum* SSBR10‐3^T^	1572	1408	1172	112
9	*Halobacillus shinanisalinarum* SSTM10‐2^T^	1572	2041	790	35
10	*Halobacillus* sp. ACCC02827	1572	1391	698	64

**FIGURE 3:**
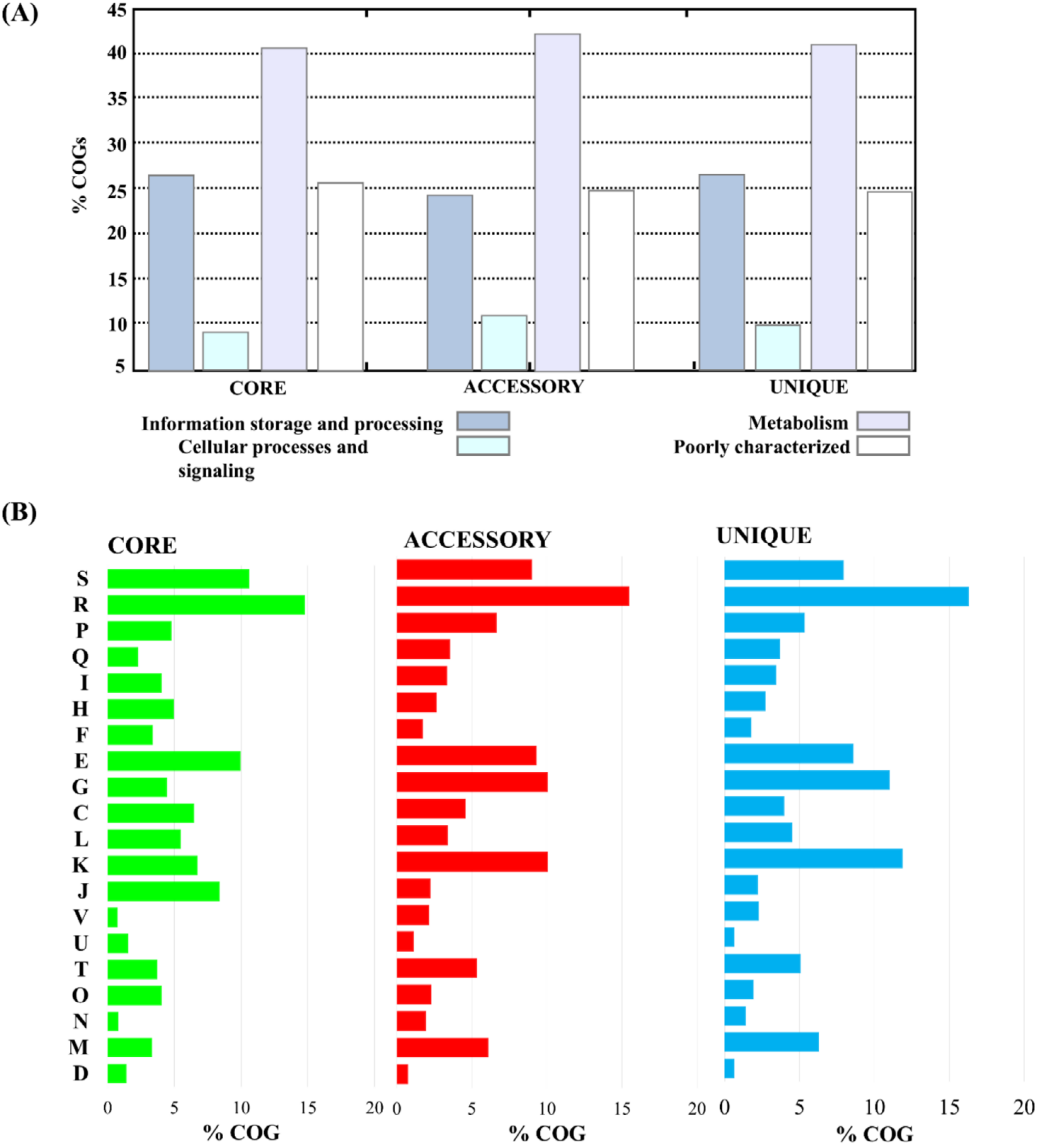
COG analysis of core, accessory, and unique genes into four major COG categories (A) and twenty subcategories (B). COG categories: R, General function prediction only; K, Transcription; G, Carbohydrate transport and metabolism; E, Amino acid transport and metabolism; S, Function unknown; V, Defence mechanisms; P, Inorganic ion transport and metabolism; M, Cell wall/membrane/envelope biogenesis; T, Signal transduction mechanisms; C, Energy production and conversion; Q, Secondary metabolites biosynthesis, transport, and catabolism; L, Replication, recombination, and repair; I, Lipid transport and metabolism; H, Coenzyme transport and metabolism; O, Post‐translational modification, protein turnover, and chaperones; J, Translation, ribosomal structure, and biogenesis; N, Cell motility; F, Nucleotide transport and metabolism; U, Intracellular trafficking, secretion, and vesicular transport; and D, Cell cycle control, cell division, and chromosome partitioning.

COG annotation from pangenome analysis categorized core, accessory, and unique genes into four major categories Figure 3A. Most genes in all three domains were related to metabolism, followed by information storage processing. This category was further divided into 20 subcategories Figure 3B. Functional analysis showed that most core genes, accessory genes, and unique gene families were only related to general function prediction (R: 14.67%, 15.37% and 16.20%). The second most prevalent category among core genes was of unknown function (S: 10.52%), The functional analysis results by COGs in ten followed by amino acid transport and metabolism (E; 9.87%). Meanwhile, transcription (K: 9.98% and 11.78%) ranked as the second most common category for accessory and unique genes, after which were carbohydrate transport and metabolism (K: 9.95% and 10.93%). The functional analysis results by COGs in ten *halobacillus* genomes showed that most core, accessory, and unique gene families were not functionally characterised, whereas most characterized genes were associated with metabolism, information storage, and processing. This finding is similar to a previous study done on salt‐tolerant bacteria *Terribacillus saccharophylus* Strain ZY‐1 (Su et al. 2022). *Halobacillus* bacteria exhibit a heightened presence of genes associated with metabolism, a trait attributed to their ability to thrive in high‐salinity environments. They undergo substantial adjustments in cellular processes to regulate osmotic balance and produce energy. They accumulate compatible solutes (Poole 1995; Roberts 2005), small molecules that do not disrupt primary metabolism to counter challenges such as water loss in saline conditions. This adaptation demands a robust metabolism to sustain the synthesis of these solutes and uphold cellular functions in high‐salt settings, which could be the reason for many genes associated with metabolism and cellular processing.

**FIGURE 4:**
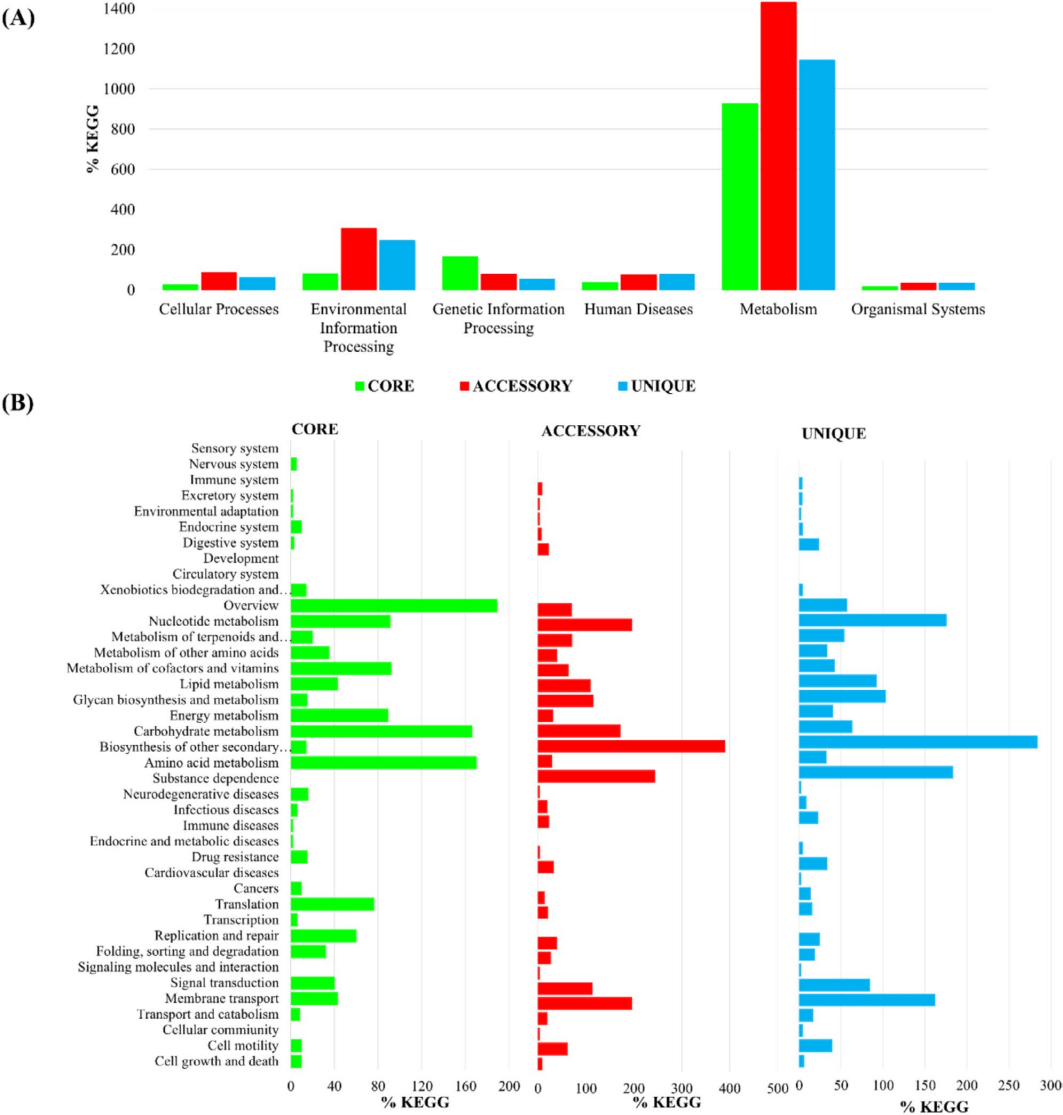
KEGG analysis of core, accessory, and unique gene into six major COG categories (A) and forty subcategories (B).

Core, accessory, and unique genes obtained from the pan‐genome analysis were further processed using the KEGG database for functional annotation. Genes were categorized into six main categories Figure 4A. Most genes were associated with metabolism, followed by environmental information processing, genetic information processing, cellular processing, human disease, and organismal system. These categories were further categorized into forty subcategories Figure 4B. Gene distribution differed in core genes compared to accessory and unique genes. In the case of core genes, most genes were attributed to overview, which might represent genes not associated with any particular category. Previously, metabolism accounted for most of the genes; hence, after subcategorizing, the highest number of genes were attributed to carbohydrate metabolism. Subsequently, amino acid metabolism had the second‐highest number of genes, which were followed by genes related to membrane transport. Carbohydrates serve as the primary energy source for bacterial growth, and the ability to metabolize various carbohydrates is vital to their survival (Agamennone et al. 2019; Liu et al. 2020). Hence, they possess a wide array of genes involved in carbohydrate metabolism. Similarly, genes associated with amino acid metabolism are essential for synthesizing, interconversion, and utilization of amino acids essential for growth and various metabolic processes (Diniz et al. 2020). A notable portion of genes also represents the xenobiotic metabolism category within the accessory, unique, and core gene categories. *Halobacillus* has been previously reported to metabolize xenobiotic compounds (Ibrahim et al. 2020; Nanca et al. 2018; Rivadeneyra et al. 2004).

### 3.4 Comparative Genomic Analysis of Features Among Three Strains of *Halobacillus* Species

**FIGURE 5:**
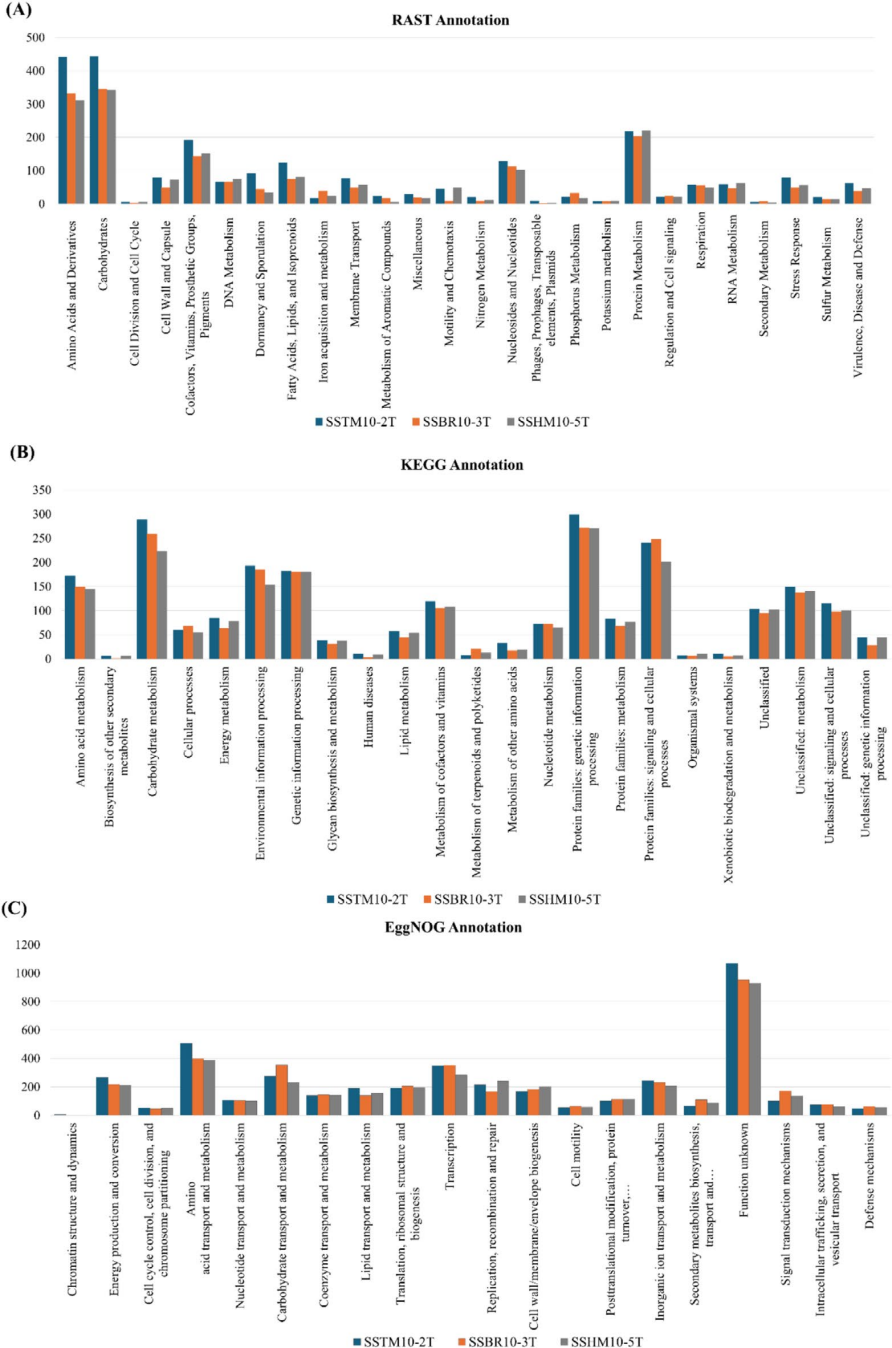
Comparative analysis of COG of strains *Halobacillus shinanisalinarum* SSTM10‐2^T^, *Halobacillus salinarum* SSBR10‐3^T^, and *Halobacillus amylolyticus* SSHM10‐5^T^ using multiple tools. (A) RAST annotation, (B) KEGG annotation, and (C) EggNOG annotation.

Functional gene annotation of three *Halobacillus* strains was conducted using three databases, with coding genes classified into distinct functional categories. The RAST annotation server identified categories such as carbohydrates, amino acids and derivatives, protein metabolism, and cofactors, along with stress‐related genes potentially supporting survival in harsh saline environments (Figure 5A). Notably, a significant number of genes associated with aromatic compounds and xenobiotic metabolism were present, aligning with the study's focus on xenobiotic degradation.

The KEGG annotation further supported the presence of numerous genes linked to xenobiotic degradation and metabolism, reinforcing the strains' potential for environmental resilience. Additionally, eggNOG‐mapper mapped the protein dataset to orthologous groups (Figure 5B), showing that amino acid metabolism (E) genes were the most prevalent in all three genomes, followed by transcription (K), carbohydrate transport and metabolism (G), energy production (C), and inorganic ion transport (P) (Figure 5C). The consistent findings across RAST, KEGG, and eggNOG annotations confirmed the reliability of these functional categorizations.

### 3.5 Identification of Genes Associated With Salt Tolerance and Stress Response in *Halobacillus* Strains SSTM10‐2^T^, SSBR10‐3^T^, and SSHM10‐5^T^

**FIGURE 6:**
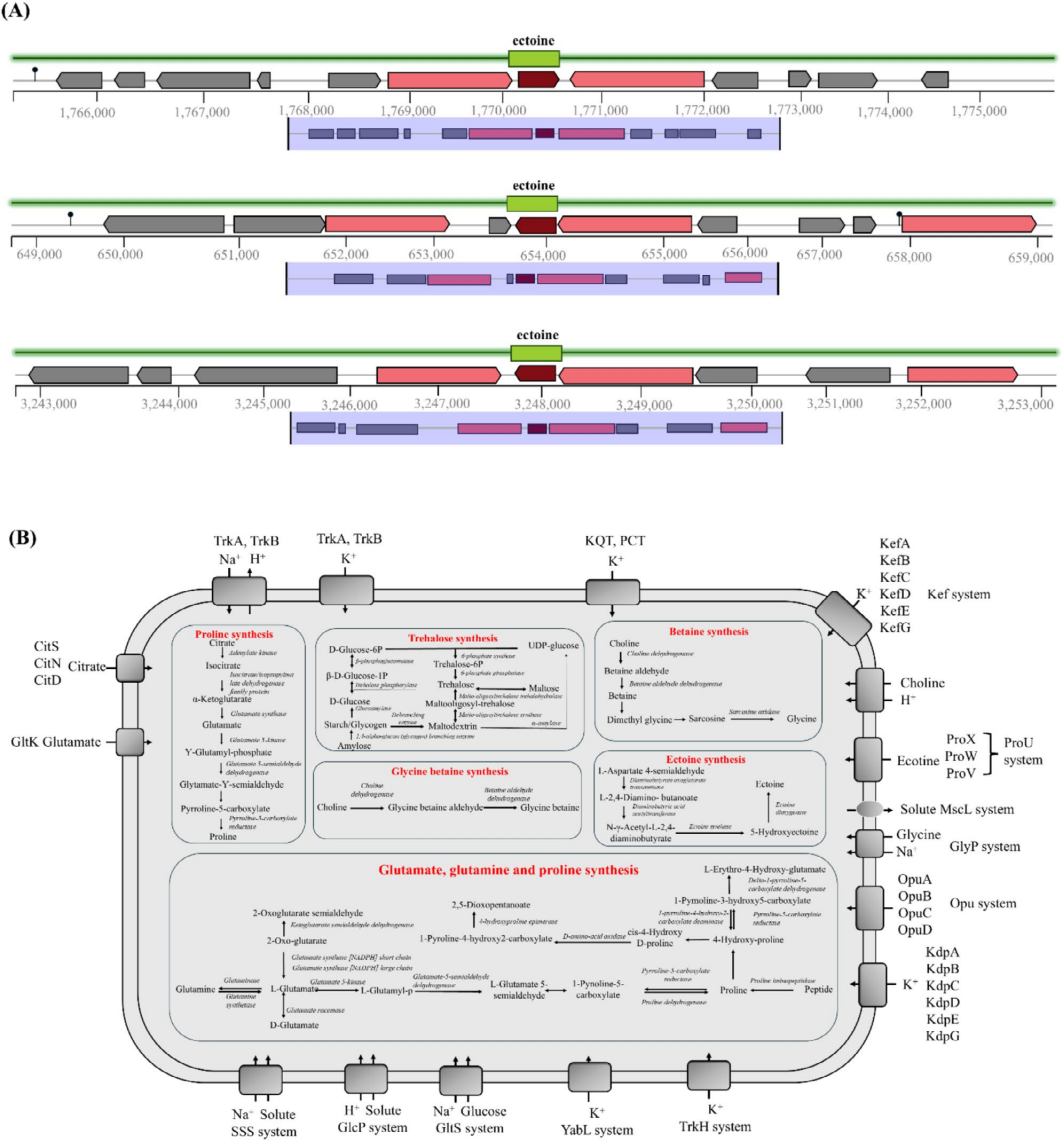
(A) Distribution of ectoine‐related BGC using antiSMASH with default settings. (B) Proposed salt adaptation pathway in *Halobacillus shinanisalinarum* SSTM10‐2^T^, *Halobacillus salinarum* SSBR10‐3^T^, and *Halobacillus amylolyticus* SSHM10‐5^T^.

The genome analysis of three *Halobacillus* strains identified key genes associated with stress tolerance, including osmotic, oxidative, and carbon starvation stress, and genes involved in stress response regulation (Supplementary Table S3). These genes enable osmoadaptation through two main strategies: the “salt‐in” strategy, which involves intracellular potassium and chloride accumulation, and the “osmolyte” strategy, which involves biosynthesis and accumulation of compatible solutes like ectoine and trehalose (Harding et al. 2016). Additionally, antiSMASH analysis revealed four biosynthetic gene clusters (BGCs) related to ectoine biosynthesis (Figure 6).

The strains also contain genes that confer resistance to heavy metals, particularly copper and fluoride exporter genes (*crcB*), which are implicated in multifaceted stress responses (Calero et al. 2022). Trehalose biosynthesis genes were identified, highlighting their role in osmotic stress tolerance, where they help to regulate reactive oxygen metabolism (ROS), photosynthesis, and ion homeostasis under high‐salinity conditions.

### 3.6 Proposed Mechanism of Algicidal Activity in *Halobacillus* Strains and Importance of This Investigation

In bacterial‐algal interactions, antagonistic effects on algae include both algicidal (causing algal death or growth inhibition) and algistatic (specifically growth‐inhibiting) effects (Dungca‐Santos et al. 2019). Recent studies suggest that *Halobacillus* may play a role in managing harmful algal blooms (HABs) through such mechanisms (Zhang et al. 2020). This research particularly focuses on *Halobacillus* strains SSTM10‐2^T^, SSBR10‐3^T^, and SSHM10‐5^T^ to explore their potential for developing chemical‐ecological methods to mitigate HABs.

#### 3.6.1 Identification of Genes Associated With CAZyme in *Halobacillus* Strains SSTM10‐2^T^ , SSBR10‐3^T^ , and SSHM10‐5^T^

**FIGURE 7:**
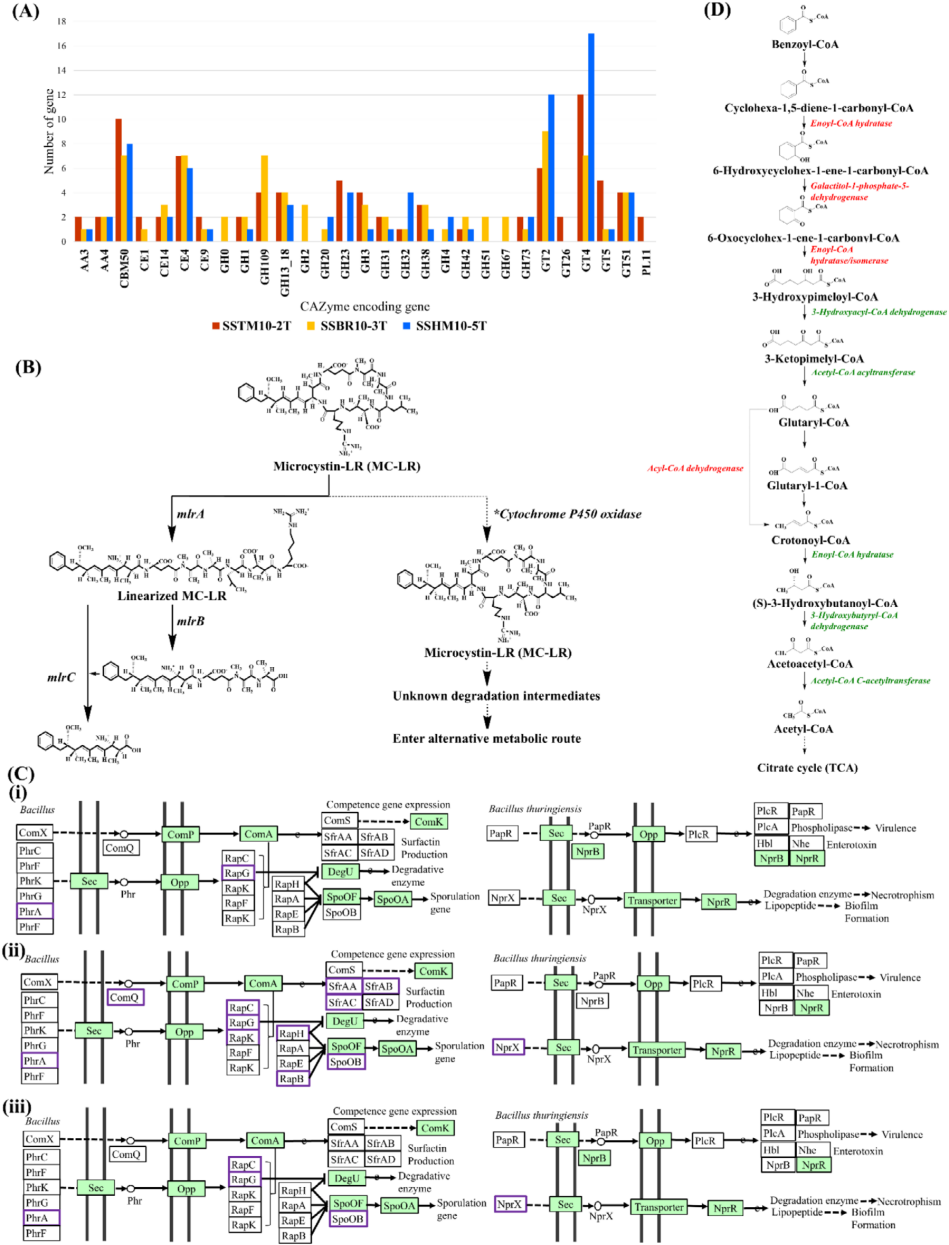
(A) The number of enzymes of each CAZyme family pattern found in genome of *Halobacillus shinanisalinarum* SSTM10‐2^T^, *Halobacillus salinarum* SSBR10‐3^T^, and *Halobacillus amylolyticus* SSHM10‐5^T^. A, auxiliary activity; CE, carbohydrate esterase; GH, glycoside hydrolase; GT, glycosyltransferase; PL, polysaccharide lyase; and CBM, carbohydrate‐binding module. (B) Proposed microcystin degradation pathway. (C) QS pathway of (i) *Halobacillus shinanisalinarum* SSTM10‐2^T^, (ii) *Halobacillus salinarum* SSBR10‐3^T^, and (iii) *Halobacillus amylolyticus* SSHM10‐5^T^ with their genetic elements. Green is from the KEGG search, and purple is from the BLAST search. (D) Aromatic compound degradation pathway of *Halobacillus shinanisalinarum* SSTM10‐2^T^, *Halobacillus salinarum* SSBR10‐3^T^, and *Halobacillus amylolyticus* SSHM10‐5^T^ with their genetic elements.

The DbCAN3‐based CAZyme analysis identified a substantial repertoire of CAZymes: 101 CAZyme genes in SSTM10‐2^T^, 105 in SSBR10‐3^T^, and 94 in SSHM10‐5^T^. These genes were classified into five prominent CAZyme families: glycoside hydrolases (GH), glycosyltransferases (GT), carbohydrate esterases (CE), auxiliary activities (AA), and carbohydrate‐binding modules (CBM). The distribution of CAZyme families is shown in Figure 7A. In each strain, glycosyltransferases (notably GT4 and GT2) were the most abundant, with SSTM10‐2^T^ exhibiting the highest number of GT4 (17) and GT2 (11) genes, followed by ce4, CBM50, and GH23 genes.

#### 3.6.2 Identification of Genes Associated With CYP Genes in *Halobacillus* Strains SSTM10‐2^T^ , SSBR10‐3^T^ , and SSHM10‐5^T^

Figure 7B shows a proposed biochemical degradation pathway for Microcystin‐LR (MC‐LR), a toxic cyclic peptide produced by cyanobacteria. MC‐LR is initially detoxified by the enzyme *mlrA*, which linearizes the compound by opening its cyclic structure—an essential step, as the cyclic form is more toxic. *mlrB* further breaks down the linearized MC‐LR into smaller peptide fragments, which are subsequently degraded by *mlrC* into non‐toxic compounds. An alternative degradation pathway may involve CYP, which is suggested to oxidize MC‐LR into intermediate products that can enter other metabolic pathways, ultimately leading to complete mineralization. Analysis of the three *Halobacillus* genomes revealed a total of 61 CYP‐related genes: 19 in SSTM10‐2^T^, 24 in SSBR10‐3^T^, and 18 in SSHM10‐5^T^—all of which map to the KEGG “Drug metabolism – CYP” pathway (Supplementary Table S4 and Figure 7B). In contrast, no other known genes associated to algicidal activities like gene encoding glutathione S‐transferases (GST) or cysteine‐conjugating enzymes were detected in any of the three genomes. Furthermore, classical *mlrA* microcystinase homologues were absent.

#### 3.6.3 Identification of Gene Associated With QS Genes in *Halobacillus* Strains SSTM10‐2^T^ , SSBR10‐3^T^ , and SSHM10‐5^T^

The gene clusters illustrated in Figure 7C (i–iii) depict a comprehensive network of competence, QS, sporulation, and virulence‐related genes across *Bacillus* species. Key QS and competence genes such as *ComX*, *ComP*, and *ComA* regulate bacterial communication and competence development. The *Rap‐Phr* cassette systems, shown prominently, function as regulatory elements that modulate gene expression in response to environmental cues, maintaining homeostasis during physiological stress. Essential for protein secretion and nutrient uptake, the *Sec* and *Opp* systems are also well represented. The *spo0A/spo0B* genes, critical to initiating the sporulation process, highlight the bacterial capacity for survival under adverse conditions. Additionally, the presence of surfactin synthesis genes (*srfAA, srfAB, srfAC*, and *srfAD*) underscores the bacteria's ability to produce biosurfactant, which is important for motility, biofilm formation, and surface colonization. On the virulence front, genes such as *nprB, plcR, papR*, and related operons produce degradative enzymes and toxins, enhancing pathogenicity through necrotrophic, phospholipase activity, and biofilm stability. Together, these systems illustrate the intricate regulatory architecture that supports environmental adaptability, community behaviour, and pathogenic potential in *Bacillus* species. The genome analysis of *Halobacillus* strains SSTM10‐2^T^, SSBR10‐3^T^, and SSHM10‐5^T^ revealed the presence of multiple genes related to the QS pathway, which was predicted through the KEGG database; green box (Figure 7C (i–iii)). Using BLAST searches against the NCBI nr database shown in the purple box (data not shown), we identified additional quorum‐sensing gene homologues whose top‐scoring matches were exclusive to *Bacillus* species—principally *B. thuringiensis* —and, to maintain consistency with our KEGG reference framework, we retained only these *Bacillus* hits for further analysis. Key genes such as *comP*, *comA*, and *comK*, which are involved in regulating competence gene expression, were found in all three strains. Moreover, genes linked to surfactin production, such as *srfAA, srfAB, and srfAC*, as well as sporulation‐related genes (*phnA*, *sec*, *opp*, *rapG*, *degU*, *spoOf*, *spoOB*, and *spoOA*), were also present. Additional genes related to degradative enzymes, biofilm formation, and virulence factors such as *sec*, *nprR*, and *nprB* were identified across the strains, suggesting that the QS mechanism might regulate algicidal activity in these *Halobacillus* strains.

#### 3.6.4 KEGG Pathways in MC‐LR Degradation and Xenobiotic Compound Degradation Associated Genes

In reference to the KEGG pathway database analysis (Mou et al. 2013), thirteen out of the 14 overexpressed KEGG pathways in microcystin‐treated microcosms observed in the metagenomic study were found in all three strains (Table 3). These pathways were primarily associated with metabolic processes such as cofactors and vitamins metabolism (22, 22, and 22 for SSTM10‐2^T^, SSBR10‐3^T^, and SSHM10‐5^T^, respectively), glycan metabolism (21, 21, and 20 for SSTM10‐2^T^, SSBR10‐3^T^, and SSHM10‐5^T^, respectively), and energy metabolism (19, 19, and 19 for SSTM10‐2^T^, SSBR10‐3^T^, and SSHM10‐5^T^, respectively). Additionally, several pathways were linked to the metabolism of xenobiotics by CYP enzymes and glutathione metabolism. The identification of pathways related to cellular processes (2 KEGGs) and environmental information processing (2 KEGGs) further suggests that these bacteria might have the potential to degrade and remove toxins produced by algae. Figure 7D presents a proposed catabolic pathway for the degradation of benzoate and other aromatic compounds in *Halobacillus* strains, supported by both KEGG pathway analysis and BLAST searches. The pathway initiates with the activation of benzoate into benzoate‐CoA, which is then converted into cyclohexa‐1,5‐diene‐1‐carbonyl‐CoA by the action of enoyl‐CoA hydratase (highlighted in red from the KEGG benzoyl‐CoA degradation pathway). Subsequent enzymatic steps involve oxidative transformations and rearrangements, progressing through intermediates like 6‐hydroxycyclohex‐1‐ene‐1‐carbonyl‐CoA and 3‐hydroxypimeloyl‐CoA. The pathway continues with the conversion of this intermediate into glutaryl‐CoA, which is then metabolized to crotonoyl‐CoA and finally acetyl‐CoA. This central metabolite feeds into the tricarboxylic acid (TCA) cycle. Enzymes identified via BLAST search are marked in green, further confirming the presence of this degradation route. This proposed pathway illustrates the metabolic versatility of *Halobacillus* strains in utilizing xenobiotic compounds.

**TABLE 3:** Significantly enriched KEGG pathways compared to KEGG pathways of MC metagenomics data.

KEGG pathway	General process	Function description	*Halobacillus shinanisalinarum* SSTM10‐2^T^	*Halobacillus salinarum* SSBR10‐3^T^	*Halobacillus amylolyticus* SSHM10‐5^T^
Cellular process					
2030	Cell motility	Bacterial chemotaxis	12	12	13
2040	Cell motility	Flagella assembly	33	33	31
Environmental information processing					
2020	Signal transduction	Two‐component system	63	63	62
3070	Membrane transport	Bacterial secretion system	11	11	11
Metabolism					
0480	Metabolism of other amino acids	Glutathione metabolism	7	7	7
0550	Glycan biosynthesis and metabolism	Peptidoglycan biosynthesis	21	21	20
0564	Lipid metabolism	Glycerophospholipid metabolism	13	13	14
0680	Energy metabolism	Methane metabolism	19	19	19
0780	Metabolism of cofactors and vitamins	Biotin metabolism	10	10	8
0860	Metabolism of cofactors and vitamins	Porphyrin and chloroplyll metabolism	22	22	21
0910	Energy Metabolism	Nitrogen metabolism	12	12	11
0920	Energy metabolism	Sulfur metabolism	16	16	18
0980	Xenobiotics biodegradation	Xenobiotics metabolism by CYP	2	2	2

## 4 Discussion

Comprehensive characterization of *Halobacillus* sp. SSTM10‐2^T^, *Halobacillus* sp. SSBR10‐3,^T^ and *Halobacillus* sp. SSHM10‐5^T^ is achieved by the construction of a circular genome map. Additionally, genomic and functional evaluation of *Halobacillus* strains SSTM10‐2^T^, SSBR10‐3^T^, and SSHM10‐5^T^ conducted in this study identified these three species as novel species. These findings were based on multiple analytical approaches like ANI, dDDH, and phylogenetic relationships. dDDH values significantly under the 70% cutoff, and ANI values falling below the 96% threshold indicated distinct taxonomic differentiation. A prior study by Kim et al. designated the strains as new species, aligning with our data. This taxonomical differentiation was further supported by the results of phylogenetic analysis, which showed that SSBR10‐3^T^ branched independently and SSTM10‐2^T^ and SSHM10‐5^T^ clustered in a separate clade. These results also show a notable genetic divergence from closely related Halobacillus species, though this is not entirely confirmed.

Genomic comparisons revealed a high degree of diversity, especially in accessory and unique genes, suggesting ongoing genetic evolution mechanisms such as mutation, gene rearrangement, or lateral gene transfer (Segerman 2012). The core genes, essential for basic metabolic functions, are highly conserved across strains, in line with their role in maintaining essential cellular processes (Jordan et al. 2002), which emphasizes the evolutionary nature of microbial genomes and their ability to adapt by incorporating non‐essential genes, especially those involved in xenobiotic metabolism (Segerman 2012). COG and KEGG pathway analysis revealed various metabolic pathways mostly related to carbohydrate and amino acid metabolism—both of which are critical for survival in saline environments (Poole 1995; Roberts 2005), hypothesizing that the bacteria carry a potential for bioremediation focusing on environmental contaminants localized within its niche (Ibrahim et al. 2020; Nanca et al. 2018; Rivadeneyra et al. 2004).

It is highly notable that dual osmoadaptation techniques, namely, “salt‐in” and “osmolyte” were found in *Halobacillus* strains. Genes involved in these techniques also synthesize ectoine and trehalose, allowing bacteria to survive in saline environments and avoid cellular damage (Brown 1976; Roberts 2005). Ectoine is well known for its role in protecting against cellular stress, in combination with trehalose, which aids in protein and membrane stabilization under various stress conditions (Sadeghi et al. 2014; Vanaporn and Titball 2020). We gathered evidence of the presence of genes associated with ectoine and trehalose gene biosynthesis from the antiSMASH result. This diverse array of stress‐related genes further provides evidence of strains capable of coping with the osmotic stress of high‐salinity environments.

One major focus of this study was to investigate the possible algicidal mechanism in *Halobacillus* species. CAZymes, particularly GH and GT, are considered to play a crucial role in breaking down algal cell walls (Han et al. 2020) were found in these three strains. This is consistent with research showing that CAZymes help break down polysaccharides in marine diatoms (Li et al. 2016). Our research applies this result to *Halobacillus*, implying that these strains may target algal polysaccharides using comparable enzymatic methods, perhaps demonstrating algicidal activity.

Our comparative genomic analysis identified 61 CYP genes across three *Halobacillus* strains. The absence of downstream conjugation enzymes like GSTs, cysteine‐conjugating enzymes, and classical *mlrA* (microcystinase) homologues suggests that *Halobacillus* may employ an alternative, CYP‐centric mechanism for MC‐LR transformation. While CYP‐mediated monooxygenation is a well‐recognized first step in MC‐LR detoxification, including epoxidation and hydroxylation reactions that increase substrate reactivity, the canonical conjugation routes that neutralize and facilitate the excretion of oxidized conjugates remain uncharacterized in these strains. The ecological significance of CYP pathways in algicidal activity extends beyond direct toxin modification. In several bacteria, CYP‐generated reactive intermediates and reactive oxygen species induce oxidative stress in algal cells, compromising membrane integrity and leading to cell lysis. However, metatranscriptomic studies of freshwater communities have thus far focused on animal detoxification models, where CYP activity correlates with glutathione and cysteine conjugation of MC‐LR (Mou et al. 2013), leaving a gap in our understanding of purely bacterial CYP‐driven algicidal mechanisms. Given that *Microcystis* spp. (notably *Microcystis aeruginosa* ) account for ~90% of HABs and are prolific MC producers (Kim et al. 2019); uncovering non‐canonical CYP pathways in *Halobacillus* could reveal novel biocontrol strategies. Nevertheless, without evidence of downstream detoxification steps, it is premature to assert algicidal efficacy. Future work should include (i) heterologous expression and biochemical characterization of identified CYPs to confirm MC‐LR oxidation kinetics, (ii) transcriptomic profiling under MC‐LR exposure to detect upregulated CYP genes, and (iii) in vitro co‐culture assays to assess bloom suppression. Such in‐depth studies will determine whether these *Halobacillus* strains represent promising candidates for bioaugmentation in mitigating cyanobacterial toxins.

Our genomic study revealed a full complement of QS‐related genes in the three *Halobacillus* strains, including those related to surfactin biosynthesis and sporulation regulation. Previous studies have shown that disrupting QS systems in other algicidal bacteria results in diminished algicidal activity (Liu et al. 2022; Wu et al. 2017), suggesting that the QS system may potentially serve as a master regulator of toxin production, exoenzyme secretion, and cell‐to‐cell coordination in algicidal contexts. Although QS pathways have not been previously characterized in *Halobacillus*, the presence of conserved signal synthase and receptor genes in their genomes suggests that these strains may also utilize QS mechanisms to coordinate and enhance their algicidal activity.

The potential relationship between xenobiotic chemical degradation and algicidal activity in *Halobacillus* is equally intriguing. Beyond the CYP monooxygenases discussed above, we detected pathways for aromatic compound degradation, hinting at a dual functionality: a direct assault on algal cells via QS‐triggered metabolites and concurrent detoxification of algal toxins or other environmental pollutants (Deng et al. 2023). While the direct causal link between xenobiotic metabolism and algicidal potency remains to be biochemically validated, the genome proximity of QS genes and catabolic gene clusters suggests a promising possibility of coordinated regulation, where toxin breakdown products might feed back into QS networks to modulate further enzyme production.

In conclusion, our study emphasizes the genomic and functional potential of three *Halobacillus* strains, SSTM10‐2^T^, SSBR10‐3^T^, and SSHM10‐5^T^, for biotechnological and environmental applications, particularly in the control of HABs. Their genomic diversity, adaptable metabolic methods, and algicidal activity indicate they could be a viable and sustainable alternative to chemical treatments in aquatic ecosystems. Future studies centred on identifying the specific enzymatic and molecular processes behind their algicidal and halotolerant action will be crucial for harnessing these strains for practical application in HAB management and bioremediation.

## Author Contributions

**Saru Gurung:** conceptualization, methodology, investigation, data curation, visualization, writing – original draft, writing – review and editing. **Chang‐Muk Lee:** conceptualization, methodology, data curation, investigation, visualization, writing – original draft, writing – review and editing. **Hang‐Yeon Weon:** methodology, data curation, writing – original draft. **So‐Ra Han:** methodology, data curation, writing – original draft. **Tae‐Jin Oh:** conceptualization, funding acquisition, project administration, resources, supervision, writing – review and editing.

## Conflicts of Interest

The authors declare no conflicts of interest.

## Supporting information


**Data S1.** Supporting Information.

## Data Availability

The data that supports the findings of this study are available in the supporting information of this article.
