# Supplementary material for: Comparative Genome Analysis of Three Halobacillus Strains Isolated From Saline Environments Reveal Potential Salt Tolerance and Algicidal Mechanisms
Source: Environ Microbiol Rep. 2025 Jun 15;17(3):e70121. doi: 10.1111/1758-2229.70121 (PMC12167742; doi:10.1111/1758-2229.70121)
Supplement: Supplementary file 1 — Data S1. Supporting Information. [file EMI4-17-e70121-s001.docx]

*Supplementary*

*data*

*for*

**Comparative genome analysis of three *Halobacillus* strains isolated from saline environments reveal potential salt tolerance and algicidal mechanisms**

Saru Gurung^1,2*^, Chang-Muk Lee^3,4*^, Hang-Yeon Weon^3^, So-Ra Han^1,2,5^ and Tae-Jin Oh^1,2,5,6^**^¶^**

^1^Department of Life Science and Biochemical Engineering, SunMoon University, Asan 31460, Republic of Korea

^2^Bio Big Data-based Chungnam Smart Clean Research Leader Training Program, SunMoon University, 31460 Asan, Republic of Korea

^3^Agricultural Microbiology Division, National Institute of Agricultural Sciences, Rural Development Administration, Jeonju 55365, Republic of Korea

^4^Technology Services Division, National Institute of Agricultural Sciences, Rural Development Administration, Jeonju 55365, Republic of Korea

^5^Genome-based BioIT Convergence Institute, Asan 31460, Republic of Korea

^6^Department of Pharmaceutical Engineering and Biotechnology, SunMoon University, Asan 31460, Republic of Korea

**Supplementary Table S1** : Feature comparison of 10 *Halobacillus* strains, complete genome.

| **S.N** | ***Halobacillus* genomes** | **Source of isolation** | **Assembly level** | **Genome size (bp)** | **Number of plasmid** | **CDS (Total)** | **GC content (mol %)** | **tRNA** | **rRNA** | **RefSeq** |
| --- | --- | --- | --- | --- | --- | --- | --- | --- | --- | --- |
| 1 | *Halobacillus shinanisalinarum* SSTM10-2^T^ | Saltern | Complete | 4,887,025 | 0 | 4,776 | 40.50 | 69 | 27 | NZ_CP095074.1 |
| 2 | *Halobacillus salinarum* SSBR10-3^T^ | Saltern | Complete | 4,240,228 | 0 | 4,265 | 42.50 | 66 | 21 | NZ_CP095073.1 |
| 3 | *Halobacillus amylolyticus* SSHM10-5^T^ | Saltern | Complete | 4,154,097 | 1 | 4,244 | 40.50 | 70 | 24 | NZ_CP095075.1 |
| 4 | *Halobacillus* *halophilus* DSM2266^T^ | N/A | Complete | 4,150,632 | 2 | 4,025 | 41.80 | 67 | 21 | NC_017668 |
| 5 | *Halobacillus* *mangrovi* KTB131 | N/A | Complete | 4,142,697 | 0 | 4,014 | 41.60 | 68 | 21 | NZ_CP020772.1 |
| 6 | *Halobacillus* *litoralis* ERB031 | Salt lake | Complete | 4,145,028 | 1 | 4,278 | 42.19 | 69 | 24 | NZ_CP026118.1 |
| 7 | *Halobacillus* naozhouensis KCTC13234 | N/A | Complete | 4,219,438 | 1 | 4,219 | 47.09 | 68 | 21 | NZ_CP121671.1 |
| 8 | *Halobacillus halophilus* HL2HP6 | Halophyte Rhizosphere | Complete | 4,148,997 | 2 | 4,033 | 41.80 | 67 | 21 | NZ_CP022106.1 |
| 9 | *Halobacillus* sp. ACCC02827 | Soil | Complete | 3,676,902 | 2 | 3,745 | 47.09 | 67 | 24 | NZ_CP128168.1 |
| 10 | *Halobacillus litoralis* SaN21-8 |  | Complete | 3,899,858 | 1 | 3,961 | 43.57 | 69 | 24 | NZ_CP129016.1 |

**Supplementary Table S2** : Pairwise digital DNA-DNA hybridization values between query genomes and seven whole genomes of *Halobacillus* strains.

| **Query genome** | **Reference genome** | **DDH** | **Model C.I. (%)** | **Distance** | **Prob. DDH >= 70%** | **G+C difference** |
| --- | --- | --- | --- | --- | --- | --- |
| *Halobacillus* *shinanisalinarum* SSTM10-2^T^ | *Halobacillus salinarum* SSBR10-3^T^ | 14.1 | [11.7 - 16.9] | 0.9467 | 0 | 1.53 |
|  | *Halobacillus amylolyticus* SSHM10-5^T^ | 49.3 | [46.2 - 52.3] | 0.3630 | 2.33 | 0.10 |
|  | *Halobacillus halophilus* HL2HP6 | 14.3 | [11.9 - 17.1] | 0.9378 | 0 | 1.10 |
|  | *Halobacillus halophilus* DSM2266^T^ | 14.3 | [11.9 - 17.1] | 0.9377 | 0 | 1.09 |
|  | *Halobacillus litoralis* ERB031 | 14.2 | [11.8 - 17.1] | 0.9402 | 0 | 1.66 |
|  | *Halobacillus mangrov* KTB131 | 14.3 | [11.8 - 17.1] | 0.9389 | 0 | 0.83 |
|  | *Halobacillus naozhouensis* KCTC13234 | 22.3 | [19.5 - 25.4] | 0.7000 | 0 | 0.74 |
|  | *Halobacillus* sp. ACCC02827 | 14.0 | [11.6 - 16.8] | 0.9503 | 0 | 6.37 |
|  | *Halobacillus* *litoralis* SaN21-8 | 13.8 | [11.4 - 16.6] | 0.9602 | 0 | 2.90 |
| *Halobacillus* *salinarum* SSBR10-3^T^ | *Halobacillus halophilus* DSM 2266^T^ | 14.7 | [12.2 - 17.5] | 0.9233 | 0 | 0.43 |
|  | *Halobacillus litoralis* ERB031 | 14.6 | [12.1 - 17.4] | 0.9262 | 0 | 0.13 |
|  | *Halobacillus mangrov* KTB131 | 14.7 | [12.2 - 17.5] | 0.9221 | 0 | 0.70 |
|  | *Halobacillus naozhouensis* KCTC13234 | 14.3 | [11.9 - 17.1] | 0.9384 | 0 | 0.79 |
|  | *Halobacillus* sp. ACCC02827 | 14.3 | [11.9 - 17.1] | 0.9370 | 0 | 4.84 |
|  | *Halobacillus shinanisalinarum* SSTM10-2^T^ | 14.1 | [11.7 - 16.9] | 0.9467 | 0 | 1.53 |
|  | *Halobacillus amylolyticus* SSHM10-5^T^ | 14.2 | [11.8 – 17.0] | 0.9427 | 0 | 1.43 |
|  | *Halobacillus halophilus* HL2HP6 | 14.7 | [12.2 - 17.5] | 0.9234 | 0 | 0.43 |
|  | Halobacillus litoralis SaN21-8 | 14.4 | [12.0 - 17.2] | 0.9331 | 0 | 1.37 |
| *Halobacillus* *amylolyticus* SSHM10-5^T^ | *Halobacillus litoralis* ERB031 | 14.2 | [11.8 - 17.1] | 0.9402 | 0 | 1.66 |
|  | *Halobacillus mangrov* KTB131 | 14.3 | [11.8 - 17.1] | 0.9389 | 0 | 0.83 |
|  | *Halobacillus naozhouensis* KCTC13234 | 22.3 | [19.5 - 25.4] | 0.7000 | 0 | 0.74 |
|  | *Halobacillus* sp. ACCC02827 | 14.0 | [11.6 - 16.8] | 0.9503 | 0 | 6.37 |
|  | *Halobacillus salinarum* SSBR10-3T | 14.1 | [11.7 - 16.9] | 0.9467 | 0 | 1.53 |
|  | *Halobacillus amylolyticus* SSHM10-5 | 49.3 | [46.2 - 52.3] | 0.3630 | 2.33 | 0.10 |
|  | *Halobacillus halophilus* HL2HP6 | 14.3 | [11.9 - 17.1] | 0.9378 | 0 | 1.10 |
|  | Halobacillus litoralis SaN21-8 | 14.1 | [11.7 - 16.9] | 0.9453 | 0 | 2.80 |
|  | *Halobacillus halophilus* DSM2266^T^ | 14.3 | [11.9 - 17.1] | 0.9377 | 0 | 1.09 |

**Supplementary Table S3** : Stress response and salt-tolerance related genes strains SSTM10-2^T^, SSBR10-3^T^, and SSHM10-5^T^.

| **SSTM10-2^T^** | | |
| --- | --- | --- |
| **Category** | **Location** | **Function** |
| Osmotic stress | CP095074.1_3123124_3124590 | Betaine aldehyde dehydrogenase (EC 1.2.1.8) |
|  | CP095074.1_3192567_3192070 | Betaine aldehyde dehydrogenase (EC 1.2.1.8) |
|  | CP095074.1_3193541_3192564 | Betaine aldehyde dehydrogenase (EC 1.2.1.8) |
|  | CP095074.1_3509322_3510800 | Betaine aldehyde dehydrogenase (EC 1.2.1.8) |
|  | CP095074.1_3124626_3126317 | Choline dehydrogenase (EC 1.1.99.1) |
|  | CP095074.1_3510970_3512652 | Choline dehydrogenase (EC 1.1.99.1) |
|  | CP095074.1_19617_20339 | Glycine betaine ABC transport system, permease protein OpuAB / Glycine betaine ABC transport system, glycine betaine-binding protein OpuAC |
|  | CP095074.1_20378_21142 | Glycine betaine ABC transport system, permease protein OpuAB / Glycine betaine ABC transport system, glycine betaine-binding protein OpuAC |
|  | CP095074.1_905387_904488 | Glycine betaine ABC transport system, glycine betaine-binding protein OpuAC |
|  | CP095074.1_3508312_3507428 | Glycine betaine ABC transport system, glycine betaine-binding protein OpuAC |
|  | CP095074.1_4129955_4130884 | Glycine betaine ABC transport system, glycine betaine-binding protein OpuAC |
|  | CP095074.1_1558721_1557147 | Glycine betaine transporter OpuD |
|  | CP095074.1_1870070_1871620 | Glycine betaine transporter OpuD |
|  | CP095074.1_2409999_2408479 | Glycine betaine transporter OpuD |
|  | CP095074.1_2863657_2862164 | Glycine betaine transporter OpuD |
|  | CP095074.1_3172774_3174354 | Glycine betaine transporter OpuD |
|  | CP095074.1_3196319_3196972 | Glycine betaine transporter OpuD |
|  | CP095074.1_3197011_3197874 | Glycine betaine transporter OpuD |
|  | CP095074.1_4053582_4055105 | Glycine betaine transporter OpuD |
|  | CP095074.1_4434500_4432962 | Glycine betaine transporter OpuD |
|  | CP095074.1_1622954_1621959 | L-proline glycine betaine ABC transport system permease protein ProV (TC 3.A.1.12.1) |
|  | CP095074.1_936406_935576 | Glycerol uptake facilitator protein |
| Oxidative stress | CP095074.1_215573_215109 | Ferric uptake regulation protein FUR |
|  | CP095074.1_2892956_2893372 | Organic hydroperoxide resistance protein |
|  | CP095074.1_3975164_3975601 | Peroxide stress regulator PerR, FUR family |
|  | CP095074.1_323120_322506 | Superoxide dismutase [Mn] (EC 1.15.1.1) |
|  | CP095074.1_77421_76921 | Superoxide dismutase [Cu-Zn] precursor (EC 1.15.1.1) |
|  | CP095074.1_840136_839567 | Superoxide dismutase [Cu-Zn] precursor (EC 1.15.1.1) |
| Bacterial haemoglobins | CP095074.1_4212867_4212466 | Hemoglobin-like protein HbO |
|  | CP095074.1_1002189_1000450 | Diguanylate cyclase/phosphodiesterase (GGDEF & EAL domains) with PAS/PAC sensor(s) |
|  | CP095074.1_2139078_2138551 | Diguanylate cyclase/phosphodiesterase (GGDEF & EAL domains) with PAS/PAC sensor(s) |
|  | CP095074.1_2756270_2755542 | Diguanylate cyclase/phosphodiesterase (GGDEF & EAL domains) with PAS/PAC sensor(s) |
|  | CP095074.1_2826616_2828085 | Diguanylate cyclase/phosphodiesterase (GGDEF & EAL domains) with PAS/PAC sensor(s) |
|  | CP095074.1_3848986_3850320 | Diguanylate cyclase/phosphodiesterase (GGDEF & EAL domains) with PAS/PAC sensor(s) |
|  | CP095074.1_3850317_3851138 | Diguanylate cyclase/phosphodiesterase (GGDEF & EAL domains) with PAS/PAC sensor(s) |
| Carbon starvation | CP095074.1_2078884_2080617 | Carbon starvation protein A |
|  | CP095074.1_2887679_2889118 | Carbon starvation protein A |
|  | CP095074.1_3331970_3333409 | Carbon starvation protein A |
|  | CP095074.1_1031796_1031569 | Carbon storage regulator |
| Dimethylarginine metabolism | CP095074.1_3046144_3047097 | NG,NG-dimethylarginine dimethylaminohydrolase 1 (EC 3.5.3.18) |
|  | CP095074.1_3292932_3292387 | NG,NG-dimethylarginine dimethylaminohydrolase 1 (EC 3.5.3.18) |
|  | CP095074.1_3651891_3652760 | NG,NG-dimethylarginine dimethylaminohydrolase 1 (EC 3.5.3.18) |
|  | CP095074.1_3285058_3284471 | Ornithine aminotransferase (EC 2.6.1.13) |
|  | CP095074.1_3285672_3285061 | Ornithine aminotransferase (EC 2.6.1.13) |
|  | CP095074.1_4171194_4170001 | Ornithine aminotransferase (EC 2.6.1.13) |
| Hfl operon | CP095074.1_4833009_4834241 | Ribosome LSU-associated GTP-binding protein HflX |
|  | CP095074.1_4831623_4831847 | RNA-binding protein Hfq |
| SigmaB stress response regulation | CP095074.1_2942269_2942670 | Anti-sigma B factor RsbT |
|  | CP095074.1_2943761_2944090 | Anti-sigma B factor antagonist RsbV |
|  | CP095074.1_2944541_2945320 | RNA polymerase sigma factor SigB |
|  | CP095074.1_1570994_1570536 | RsbR, positive regulator of sigma-B |
|  | CP095074.1_2075763_2074933 | RsbR, positive regulator of sigma-B |
|  | CP095074.1_2720109_2719261 | RsbR, positive regulator of sigma-B |
|  | CP095074.1_2941910_2942266 | RsbS, negative regulator of sigma-B |
|  | CP095074.1_2942687_2943697 | Serine phosphatase RsbU, regulator of sigma subunit |
|  | CP095074.1_2944093_2944569 | Serine-protein kinase RsbW (EC 2.7.11.1) |
| Amino acids and derivatives | CP095074.1_168662_167391 | NAD-specific glutamate dehydrogenase (EC 1.4.1.2) |
|  | CP095074.1_236239_234905 | NAD-specific glutamate dehydrogenase (EC 1.4.1.2); NADP-specific glutamate dehydrogenase (EC 1.4.1.4) |
|  | CP095074.1_3010908_3011864 | NAD-specific glutamate dehydrogenase (EC 1.4.1.2); NADP-specific glutamate dehydrogenase (EC 1.4.1.4) |
|  | CP095074.1_3284434_3283142 | NAD-specific glutamate dehydrogenase (EC 1.4.1.2) |
|  | CP095074.1_3751558_3752934 | NAD-specific glutamate dehydrogenase (EC 1.4.1.2); NADP-specific glutamate dehydrogenase (EC 1.4.1.4) |
|  | CP095074.1_530864_530106 | Glutamate racemase (EC 5.1.1.3) |
|  | CP095074.1_1428772_1427288 | Glutamate synthase [NADPH] small chain (EC 1.4.1.13) |
|  | CP095074.1_1432035_1428793 | Glutamate synthase [NADPH] large chain (EC 1.4.1.13) |
|  | CP095074.1_1432436_1432041 | Glutamate synthase [NADPH] large chain (EC 1.4.1.13) |
|  | CP095074.1_1433398_1432433 | Glutamate synthase [NADPH] large chain (EC 1.4.1.13) |
|  | CP095074.1_2221843_2221340 | Glutamate synthase [NADPH] small chain (EC 1.4.1.13) |
|  | CP095074.1_2222826_2221870 | Glutamate synthase [NADPH] small chain (EC 1.4.1.13) |
|  | CP095074.1_2227443_2222851 | Glutamate synthase [NADPH] large chain (EC 1.4.1.13) |
|  | CP095074.1_2766613_2765672 | Glutaminase (EC 3.5.1.2) |
|  | CP095074.1_4445058_4445990 | Glutaminase (EC 3.5.1.2) |
|  | CP095074.1_3834872_3833562 | Glutamine synthetase type I (EC 6.3.1.2) |
|  | CP095074.1_4836057_4837388 | Glutamine synthetase type I (EC 6.3.1.2) |
|  | CP095074.1_1316315_1314768 | Delta-1-pyrroline-5-carboxylate dehydrogenase (EC 1.2.1.88) |
|  | CP095074.1_3249646_3251196 | Delta-1-pyrroline-5-carboxylate dehydrogenase (EC 1.2.1.88) |
|  | CP095074.1_3251698_3253209 | Proline/sodium symporter PutP (TC 2.A.21.2.1) @ Propionate/sodium symporter |
|  | CP095074.1_3819316_3820578 | Gamma-glutamyl phosphate reductase (EC 1.2.1.41) |
|  | CP095074.1_3818120_3819253 | Glutamate 5-kinase (EC 2.7.2.11) / RNA-binding C-terminal domain PUA |
|  | CP095074.1_3817220_3818062 | Pyrroline-5-carboxylate reductase (EC 1.5.1.2) |
|  | CP095074.1_4007096_4007911 | Pyrroline-5-carboxylate reductase (EC 1.5.1.2) |
| Iron acquisition and metabolism | CP095074.1_2734829_2735584 | Uncharacterized iron compound ABC uptake transporter, ATP-binding protein |
|  | CP095074.1_2732945_2733898 | Uncharacterized iron compound ABC uptake transporter, permease protein |
|  | CP095074.1_2733888_2734832 | Uncharacterized iron compound ABC uptake transporter, permease protein |
|  | CP095074.1_2735608_2736540 | Iron compound ABC uptake transporter substrate-binding protein |
|  | CP095074.1_2339900_2339196 | Two-component response regulator SA14-24 |
|  | CP095074.1_2338770_2337352 | Two-component sensor kinase SA14-24 |
|  | CP095074.1_2339189_2338857 | Two-component sensor kinase SA14-24 |
|  | CP095074.1_3320064_3321143 | Two-component sensor kinase SA14-24 |
|  | CP095074.1_2335129_2334335 | Zn-dependent hydrolase YycJ/WalJ, required for cell wall metabolism and coordination of cell division with DNA replication |
|  | CP095074.1_2277141_2277422 | Heme efflux system permease HrtB |
|  | CP095074.1_2277478_2278176 | Heme efflux system permease HrtB |
|  | CP095074.1_2275846_2277066 | Sensor histidine kinase colocalized with HrtAB transporter |
|  | CP095074.1_2275027_2275695 | Two-component response regulator colocalized with HrtAB transporter |
| Potassium homeostasis | CP095074.1_2773374_2773967 | Large-conductance mechanosensitive channel |
|  | CP095074.1_2363289_2362405 | Potassium efflux system KefA protein / Small-conductance mechanosensitive channel |
|  | CP095074.1_3867126_3866266 | Potassium efflux system KefA protein / Small-conductance mechanosensitive channel |
|  | CP095074.1_4411795_4410950 | Potassium efflux system KefA protein / Small-conductance mechanosensitive channel |
|  | CP095074.1_792796_791771 | Potassium voltage-gated channel subfamily KQT; possible potassium channel, VIC family |
|  | CP095074.1_3840006_3839338 | Potassium voltage-gated channel subfamily KQT; possible potassium channel, VIC family |
|  | CP095074.1_832966_833964 | potassium channel protein |
|  | CP095074.1_3972228_3972638 | Potassium channel protein |
| Fatty acids | CP095074.1_1692332_1691910 | 3-Hydroxyacyl-[acyl-carrier-protein] dehydratase, FabZ form (EC 4.2.1.59) |
|  | CP095074.1_715557_714595 | Acetyl-coenzyme A carboxyl transferase alpha chain (EC 6.4.1.2) |
|  | CP095074.1_716414_715554 | Acetyl-coenzyme A carboxyl transferase beta chain (EC 6.4.1.2) |
|  | CP095074.1_240862_239312 | Acetyl-coenzyme A carboxyl transferase alpha chain (EC 6.4.1.2) / Acetyl-coenzyme A carboxyl transferase beta chain (EC 6.4.1.2); Propionyl-CoA carboxylase beta chain (EC 6.4.1.3) |
|  | CP095074.1_2684446_2683985 | Acetyl-coenzyme A carboxyl transferase alpha chain (EC 6.4.1.2) / Acetyl-coenzyme A carboxyl transferase beta chain (EC 6.4.1.2); Propionyl-CoA carboxylase beta chain (EC 6.4.1.3) |
|  | CP095074.1_4615009_4615242 | Acyl carrier protein |
|  | CP095074.1_275252_273879 | Biotin carboxylase of acetyl-CoA carboxylase (EC 6.3.4.14) |
|  | CP095074.1_275762_275268 | Biotin carboxyl carrier protein of acetyl-CoA carboxylase |
|  | CP095074.1_4220168_4220944 | Enoyl-[acyl-carrier-protein] reductase [NADH] (EC 1.3.1.9) |
|  | CP095074.1_4373910_4374689 | Enoyl-[acyl-carrier-protein] reductase [NADH] (EC 1.3.1.9) |
|  | CP095074.1_3603004_3603933 | Malonyl CoA-acyl carrier protein transacylase (EC 2.3.1.39) |
|  | CP095074.1_4613268_4614209 | Malonyl CoA-acyl carrier protein transacylase (EC 2.3.1.39) |
|  | CP095074.1_894991_893816 | 3-Ketoacyl-CoA thiolase [fadN-fadA-fadE operon] (EC 2.3.1.16) |
|  | CP095074.1_1276455_1275271 | 3-Ketoacyl-CoA thiolase (EC 2.3.1.16) @ Acetyl-CoA acetyltransferase (EC 2.3.1.9) |
|  | CP095074.1_1305871_1304801 | 3-Ketoacyl-CoA thiolase (EC 2.3.1.16) @ Acetyl-CoA acetyltransferase (EC 2.3.1.9) |
|  | CP095074.1_1305969_1305868 | 3-Ketoacyl-CoA thiolase (EC 2.3.1.16) @ Acetyl-CoA acetyltransferase (EC 2.3.1.9) |
|  | CP095074.1_1899127_1897919 | 3-Ketoacyl-CoA thiolase (EC 2.3.1.16) @ Acetyl-CoA acetyltransferase (EC 2.3.1.9) |
|  | CP095074.1_2191006_2190539 | 3-Ketoacyl-CoA thiolase (EC 2.3.1.16) @ Acetyl-CoA acetyltransferase (EC 2.3.1.9) |
|  | CP095074.1_2191692_2191030 | 3-Ketoacyl-CoA thiolase (EC 2.3.1.16) @ Acetyl-CoA acetyltransferase (EC 2.3.1.9) |
|  | CP095074.1_2248995_2247844 | 3-Ketoacyl-CoA thiolase (EC 2.3.1.16) @ Acetyl-CoA acetyltransferase (EC 2.3.1.9) |
|  | CP095074.1_3161187_3162371 | 3-Ketoacyl-CoA thiolase (EC 2.3.1.16) @ Acetyl-CoA acetyltransferase (EC 2.3.1.9) |
|  | CP095074.1_3773955_3775154 | 3-Ketoacyl-CoA thiolase (EC 2.3.1.16) @ Acetyl-CoA acetyltransferase (EC 2.3.1.9) |
|  | CP095074.1_3933296_3934450 | 3-Ketoacyl-CoA thiolase (EC 2.3.1.16) @ Acetyl-CoA acetyltransferase (EC 2.3.1.9) |
|  | CP095074.1_4581329_4582516 | 3-Ketoacyl-CoA thiolase (EC 2.3.1.16) @ Acetyl-CoA acetyltransferase (EC 2.3.1.9) |
|  | CP095074.1_670036_669263 | Enoyl-CoA hydratase (EC 4.2.1.17) |
|  | CP095074.1_1274245_1273325 | Enoyl-CoA hydratase (EC 4.2.1.17) |
|  | CP095074.1_1612101_1611295 | Enoyl-CoA hydratase (EC 4.2.1.17) |
|  | CP095074.1_1897804_1897025 | Enoyl-CoA hydratase (EC 4.2.1.17) |
|  | CP095074.1_1907418_1906855 | Enoyl-CoA hydratase (EC 4.2.1.17) |
|  | CP095074.1_3761217_3760441 | Enoyl-CoA hydratase (EC 4.2.1.17) |
|  | CP095074.1_3769958_3770731 | Enoyl-CoA hydratase (EC 4.2.1.17) |
|  | CP095074.1_3770735_3771517 | Enoyl-CoA hydratase (EC 4.2.1.17) |
|  | CP095074.1_3831672_3832460 | Enoyl-CoA hydratase (EC 4.2.1.17) |
|  | CP095074.1_4093059_4093829 | Enoyl-CoA hydratase (EC 4.2.1.17) |
|  | CP095074.1_4472398_4472841 | Enoyl-CoA hydratase (EC 4.2.1.17) |
|  | CP095074.1_4563877_4564662 | Enoyl-CoA hydratase (EC 4.2.1.17) |
|  | CP095074.1_4579372_4580151 | Enoyl-CoA hydratase (EC 4.2.1.17) |
|  | CP095074.1_28439_30079 | Long-chain-fatty-acid--CoA ligase (EC 6.2.1.3) |
|  | CP095074.1_672439_670730 | Long-chain-fatty-acid--CoA ligase (EC 6.2.1.3) |
|  | CP095074.1_779603_778011 | Long-chain-fatty-acid--CoA ligase (EC 6.2.1.3) |
|  | CP095074.1_1150198_1149269 | Long-chain-fatty-acid--CoA ligase (EC 6.2.1.3) |
|  | CP095074.1_1282949_1281318 | Long-chain-fatty-acid--CoA ligase (EC 6.2.1.3) |
|  | CP095074.1_1302554_1300875 | Long-chain-fatty-acid--CoA ligase (EC 6.2.1.3) |
|  | CP095074.1_2189633_2188077 | Long-chain-fatty-acid--CoA ligase (EC 6.2.1.3) |
|  | CP095074.1_2250465_2248996 | Long-chain-fatty-acid--CoA ligase (EC 6.2.1.3) |
|  | CP095074.1_3106378_3107868 | Long-chain-fatty-acid--CoA ligase (EC 6.2.1.3) |
|  | CP095074.1_3188849_3190459 | Long-chain-fatty-acid--CoA ligase (EC 6.2.1.3) |
|  | CP095074.1_4149725_4151257 | Long-chain-fatty-acid--CoA ligase (EC 6.2.1.3) |
|  | CP095074.1_4340795_4341631 | 3-Oxoacyl-[acyl-carrier protein] reductase (EC 1.1.1.100) |
|  | CP095074.1_4614209_4614949 | 3-Oxoacyl-[acyl-carrier protein] reductase (EC 1.1.1.100), FadG |
|  | CP095074.1_4713989_4714708 | 3-Oxoacyl-[acyl-carrier protein] reductase (EC 1.1.1.100) |
| Protein folding | CP095074.1_4339_5043 | Cytochrome c-type biogenesis protein CcdA (DsbD analog) |
|  | CP095074.1_626751_626041 | Cytochrome c-type biogenesis protein CcdA (DsbD analog) |
|  | CP095074.1_1441544_1440801 | Cytochrome c-type biogenesis protein CcdA (DsbD analog) |
|  | CP095074.1_360678_359554 | Chaperone protein DnaJ |
|  | CP095074.1_362650_360821 | Chaperone protein DnaK |
|  | CP095074.1_363235_362687 | Heat shock protein GrpE |
|  | CP095074.1_200937_200575 | Peptidyl-prolyl cis-trans isomerase (EC 5.2.1.8) |
| Protein processing and modification | CP095074.1_2546027_2546524 | SSU ribosomal protein S5p (S2e) |
|  | CP095074.1_4510056_4510529 | Lipoprotein signal peptidase (EC 3.4.23.36) |
|  | CP095074.1_1346635_1346075 | Signal peptidase SipW (EC 3.4.21.89), required for TasA secretion |
|  | CP095074.1_3428219_3428797 | Signal peptidase SipW (EC 3.4.21.89), required for TasA secretion |
|  | CP095074.1_4154195_4154734 | Signal peptidase I (EC 3.4.21.89) |
|  | CP095074.1_4396973_4396452 | Signal peptidase I (EC 3.4.21.89) |
|  | CP095074.1_4627139_4627687 | Signal peptidase I (EC 3.4.21.89) |
|  | CP095074.1_1820166_1819705 | Lipid carrier : UDP-N-acetylgalactosaminyltransferase (EC 2.4.1.-) |
| Cation transporter | CP095074.1_616452_614047 | Lead, cadmium, zinc and mercury transporting ATPase (EC 3.6.3.3) (EC 3.6.3.5); Copper-translocating P-type ATPase (EC 3.6.3.4) |
|  | CP095074.1_622785_620701 | Lead, cadmium, zinc and mercury transporting ATPase (EC 3.6.3.3) (EC 3.6.3.5); Copper-translocating P-type ATPase (EC 3.6.3.4) |
|  | CP095074.1_1930319_1928970 | Lead, cadmium, zinc and mercury transporting ATPase (EC 3.6.3.3) (EC 3.6.3.5); Copper-translocating P-type ATPase (EC 3.6.3.4) |
|  | CP095074.1_1931509_1930271 | Lead, cadmium, zinc and mercury transporting ATPase (EC 3.6.3.3) (EC 3.6.3.5); Copper-translocating P-type ATPase (EC 3.6.3.4) |
|  | CP095074.1_1934745_1932340 | Lead, cadmium, zinc and mercury transporting ATPase (EC 3.6.3.3) (EC 3.6.3.5); Copper-translocating P-type ATPase (EC 3.6.3.4) |
|  | CP095074.1_1954223_1952157 | Lead, cadmium, zinc and mercury transporting ATPase (EC 3.6.3.3) (EC 3.6.3.5); Copper-translocating P-type ATPase (EC 3.6.3.4) |
|  | CP095074.1_3856417_3854489 | Lead, cadmium, zinc and mercury transporting ATPase (EC 3.6.3.3) (EC 3.6.3.5); Copper-translocating P-type ATPase (EC 3.6.3.4) |
|  | CP095074.1_616785_616468 | Repressor CsoR of the copZA operon |
|  | CP095074.1_1935077_1934760 | Repressor CsoR of the copZA operon |
|  | CP095074.1_614024_613821 | Copper(I) chaperone CopZ |
|  | CP095074.1_1932314_1932111 | Copper(I) chaperone CopZ |
|  | CP095074.1_4379778_4380731 | Magnesium and cobalt transport protein CorA |
| Multi-subunit cation antiporter | CP095074.1_1968182_1966311 | Na(+)/H(+) antiporter |
|  | CP095074.1_382840_385176 | Na(+) H(+) antiporter subunit A |
|  | CP095074.1_5547_5975 | Na(+) H(+) antiporter subunit B |
|  | CP095074.1_3270002_3269280 | Na(+) H(+) antiporter subunit B |
|  | CP095074.1_385180_385518 | Na(+) H(+) antiporter subunit C |
|  | CP095074.1_385511_386995 | Na(+) H(+) antiporter subunit D |
|  | CP095074.1_3268692_3267454 | Na(+) H(+) antiporter subunit D |
|  | CP095074.1_387003_387479 | Na(+) H(+) antiporter subunit E |
|  | CP095074.1_387479_387790 | Na(+) H(+) antiporter subunit F |
|  | CP095074.1_387828_388142 | Na(+) H(+) antiporter subunit G |
| Trehalose biosynthesis | CP095074.1_2052072_2051359 | Trehalose operon transcriptional repressor |
|  | CP095074.1_2053791_2052097 | Trehalose-6-phosphate hydrolase (EC 3.2.1.93) |
|  | CP095074.1_2055232_2053823 | PTS system, trehalose-specific IIB component (EC 2.7.1.201) / PTS system, trehalose-specific IIC component |
| Trehalose uptake and utilizaation | CP095074.1_1132882_1132163 | Trehalose utilization protein ThuA |
|  | CP095074.1_3526006_3526695 | Beta-phosphoglucomutase (EC 5.4.2.6) |
| Polysaccharides | CP095074.1_2262233_2260314 | 1,4-Alpha-glucan (glycogen) branching enzyme, GH-13-type (EC 2.4.1.18) |
|  | CP095074.1_2258584_2258045 | Glycogen biosynthesis protein GlgD, glucose-1-phosphate adenylyltransferase family |
|  | CP095074.1_2259150_2258791 | Glycogen biosynthesis protein GlgD, glucose-1-phosphate adenylyltransferase family |
|  | CP095074.1_2260324_2259164 | Glucose-1-phosphate adenylyltransferase (EC 2.7.7.27) |
|  | CP095073.1_626561_627721 | Glucose-1-phosphate adenylyltransferase (EC 2.7.7.27) |
|  | CP095074.1_2257750_2256311 | Glycogen synthase, ADP-glucose transglucosylase (EC 2.4.1.21) |
|  | CP095074.1_2256296_2253858 | Glycogen phosphorylase (EC 2.4.1.1) |
| **SSBR10-3^T^** | | |
| **Category** | **Location** | **Function** |
| Osmotic stress | CP095073.1_2524729_2526201 | Betaine aldehyde dehydrogenase (EC 1.2.1.8) |
|  | CP095073.1_4140827_4139361 | Betaine aldehyde dehydrogenase (EC 1.2.1.8) |
|  | CP095073.1_4139317_4137632 | Choline dehydrogenase (EC 1.1.99.1) |
|  | CP095073.1_1743157_1744053 | Glycine betaine ABC transport system, glycine betaine-binding protein OpuAC |
|  | CP095073.1_2605651_2604641 | Glycine betaine ABC transport system, permease protein OpuAB / Glycine betaine ABC transport system, glycine betaine-binding protein OpuAC |
|  | CP095073.1_2606163_2605663 | Glycine betaine ABC transport system, permease protein OpuAB / Glycine betaine ABC transport system, glycine betaine-binding protein OpuAC |
|  | CP095073.1_2520749_2519223 | Glycine betaine transporter OpuD |
|  | CP095073.1_2865193_2866725 | Glycine betaine transporter OpuD |
|  | CP095073.1_3714941_3716455 | Glycine betaine transporter OpuD |
|  | CP095073.1_4206672_4208201 | Glycine betaine transporter OpuD |
|  | CP095073.1_126182_125352 | Glycerol uptake facilitator protein |
|  | CP095073.1_1570388_1571191 | Glycerol uptake facilitator protein |
| Oxidative stress | CP095073.1_2287001_2287489 | Ferric uptake regulation protein FUR |
|  | CP095073.1_4211260_4210844 | Organic hydroperoxide resistance protein |
|  | CP095073.1_3424439_3424879 | Organic hydroperoxide resistance transcriptional regulator |
|  | CP095073.1_3423944_3424423 | Glutathione peroxidase (EC 1.11.1.9) @ Thioredoxin peroxidase (EC 1.11.1.15) |
|  | CP095073.1_3357087_3356629 | Peroxide stress regulator PerR, FUR family |
|  | CP095073.1_2142863_2143477 | Superoxide dismutase [Mn] (EC 1.15.1.1) |
|  | CP095073.1_1158597_1159268 | Predicted N-ribosylNicotinamide CRP-like regulator |
|  | CP095073.1_1799012_1799587 | Superoxide dismutase [Cu-Zn] precursor (EC 1.15.1.1) |
|  | CP095073.1_2438091_2438594 | Superoxide dismutase [Cu-Zn] precursor (EC 1.15.1.1) |
|  | CP095073.1_3765632_3766243 | Superoxide dismutase [Cu-Zn] precursor (EC 1.15.1.1) |
| Periplasmic stress | CP095073.1_2692802_2691546 | Intramembrane protease RasP/YluC, implicated in cell division based on FtsL cleavage |
| Bacterial haemoglobins | CP095073.1_3111035_3111436 | Hemoglobin-like protein HbO |
|  | CP095073.1_1288612_1286771 | Diguanylate cyclase/phosphodiesterase (GGDEF & EAL domains) with PAS/PAC sensor(s) |
| Carbon starvation | CP095073.1_1293528_1292047 | Carbon starvation protein A |
|  | CP095073.1_1293782_1293588 | Carbon starvation protein A |
|  | CP095073.1_1438004_1438231 | Carbon storage regulator |
| Dimethylarginine metabolism | CP095073.1_989313_990170 | NG,NG-dimethylarginine dimethylaminohydrolase 1 (EC 3.5.3.18) |
|  | CP095073.1_3155785_3155925 | Ornithine aminotransferase (EC 2.6.1.13) |
|  | CP095073.1_3155886_3156158 | Ornithine aminotransferase (EC 2.6.1.13) |
|  | CP095073.1_3156221_3156976 | Ornithine aminotransferase (EC 2.6.1.13) |
| Hfl operon | CP095073.1_2634398_2633163 | Ribosome LSU-associated GTP-binding protein HflX |
|  | CP095073.1_2635740_2635516 | RNA-binding protein Hfq |
| SigmaB stress response regulation | CP095073.1_1658025_1658423 | Anti-sigma B factor RsbT |
|  | CP095073.1_4172706_4172305 | Anti-sigma B factor RsbT |
|  | CP095073.1_4171219_4170890 | Anti-sigma B factor antagonist RsbV |
|  | CP095073.1_4170439_4169660 | RNA polymerase sigma factor SigB |
|  | CP095073.1_4173065_4172709 | RsbS, negative regulator of sigma-B |
|  | CP095073.1_4172292_4171282 | Serine phosphatase RsbU, regulator of sigma subunit |
|  | CP095073.1_4170887_4170414 | Serine-protein kinase RsbW (EC 2.7.11.1) |
| Amino acids and derivatives | CP095073.1_660151_660516 | NAD-specific glutamate dehydrogenase (EC 1.4.1.2) |
|  | CP095073.1_660513_661460 | NAD-specific glutamate dehydrogenase (EC 1.4.1.2); NADP-specific glutamate dehydrogenase (EC 1.4.1.4) |
|  | CP095073.1_2329046_2330326 | NAD-specific glutamate dehydrogenase (EC 1.4.1.2) |
|  | CP095073.1_3676782_3675406 | NAD-specific glutamate dehydrogenase (EC 1.4.1.2); NADP-specific glutamate dehydrogenase (EC 1.4.1.4) |
|  | CP095073.1_1979485_1980306 | Glutamate racemase (EC 5.1.1.3) |
|  | CP095073.1_638622_643205 | Glutamate synthase [NADPH] large chain (EC 1.4.1.13) |
|  | CP095073.1_643224_644711 | Glutamate synthase [NADPH] small chain (EC 1.4.1.13) |
|  | CP095073.1_76839_77810 | Glutaminase (EC 3.5.1.2) |
|  | CP095073.1_2854311_2853379 | Glutaminase (EC 3.5.1.2) |
|  | CP095073.1_2631342_2630011 | Glutamine synthetase type I (EC 6.3.1.2) |
|  | CP095073.1_2981082_2982392 | Glutamine synthetase type I (EC 6.3.1.2) |
|  | CP095073.1_1235531_1237078 | Delta-1-pyrroline-5-carboxylate dehydrogenase (EC 1.2.1.88) |
|  | CP095073.1_3840297_3838747 | Delta-1-pyrroline-5-carboxylate dehydrogenase (EC 1.2.1.88) |
|  | CP095073.1_3837623_3836100 | Proline/sodium symporter PutP (TC 2.A.21.2.1) @ Propionate/sodium symporter |
|  | CP095073.1_3600949_3599687 | Gamma-glutamyl phosphate reductase (EC 1.2.1.41) |
|  | CP095073.1_3602097_3600970 | Glutamate 5-kinase (EC 2.7.2.11) / RNA-binding C-terminal domain PUA |
|  | CP095073.1_4131940_4130837 | Glutamate 5-kinase (EC 2.7.2.11) / RNA-binding C-terminal domain PUA |
|  | CP095073.1_3309719_3308907 | Pyrroline-5-carboxylate reductase (EC 1.5.1.2) |
|  | CP095073.1_3602999_3602157 | Pyrroline-5-carboxylate reductase (EC 1.5.1.2) |
| Iron acquisition and metabolism | CP095073.1_398270_398974 | Two-component response regulator SA14-24 |
|  | CP095073.1_398982_400808 | Two-component sensor kinase SA14-24 |
|  | CP095073.1_403047_403841 | Zn-dependent hydrolase YycJ/WalJ, required for cell wall metabolism and coordination of cell division with DNA replication |
| Potassium homeostasis | CP095073.1_66568_66086 | Large-conductance mechanosensitive channel |
|  | CP095073.1_365730_366632 | Potassium efflux system KefA protein / Small-conductance mechanosensitive channel |
|  | CP095073.1_2904295_2905143 | Potassium efflux system KefA protein / Small-conductance mechanosensitive channel |
|  | CP095073.1_3507508_3506417 | Potassium efflux system KefA protein / Small-conductance mechanosensitive channel |
|  | CP095073.1_3552894_3553763 | Potassium efflux system KefA protein / Small-conductance mechanosensitive channel |
|  | CP095073.1_1845673_1846698 | Potassium voltage-gated channel subfamily KQT; possible potassium channel, VIC family |
|  | CP095073.1_3576923_3577567 | Potassium voltage-gated channel subfamily KQT; possible potassium channel, VIC family |
|  | CP095073.1_1806944_1805946 | potassium channel protein |
|  | CP095073.1_3359747_3359337 | Potassium channel protein |
| Fatty acids | CP095073.1_1020940_1021362 | 3-Hydroxyacyl-[acyl-carrier-protein] dehydratase, FabZ form (EC 4.2.1.59) |
|  | CP095073.1_1920087_1920965 | Acetyl-coenzyme A carboxyl transferase beta chain (EC 6.4.1.2) |
|  | CP095073.1_1920943_1921905 | Acetyl-coenzyme A carboxyl transferase alpha chain (EC 6.4.1.2) |
|  | CP095073.1_2260288_2261829 | Acetyl-coenzyme A carboxyl transferase alpha chain (EC 6.4.1.2) / Acetyl-coenzyme A carboxyl transferase beta chain (EC 6.4.1.2); Propionyl-CoA carboxylase beta chain (EC 6.4.1.3) |
|  | CP095073.1_2752700_2752467 | Acyl carrier protein |
|  | CP095073.1_2196033_2196389 | Biotin carboxyl carrier protein of acetyl-CoA carboxylase |
|  | CP095073.1_2196401_2196535 | Biotin carboxyl carrier protein of acetyl-CoA carboxylase |
|  | CP095073.1_2196551_2197909 | Biotin carboxylase of acetyl-CoA carboxylase (EC 6.3.4.14) |
|  | CP095073.1_2595950_2594595 | Biotin carboxylase of acetyl-CoA carboxylase (EC 6.3.4.14) |
|  | CP095073.1_3103482_3102706 | Enoyl-[acyl-carrier-protein] reductase [NADH] (EC 1.3.1.9) |
|  | CP095073.1_3599013_3598234 | Enoyl-[acyl-carrier-protein] reductase [NADH] (EC 1.3.1.9) |
|  | CP095073.1_2754442_2753501 | Malonyl CoA-acyl carrier protein transacylase (EC 2.3.1.39) |
|  | CP095073.1_572428_573618 | 3-Ketoacyl-CoA thiolase (EC 2.3.1.16) @ Acetyl-CoA acetyltransferase (EC 2.3.1.9) |
|  | CP095073.1_663662_664813 | 3-Ketoacyl-CoA thiolase (EC 2.3.1.16) @ Acetyl-CoA acetyltransferase (EC 2.3.1.9) |
|  | CP095073.1_706141_707295 | 3-Ketoacyl-CoA thiolase (EC 2.3.1.16) @ Acetyl-CoA acetyltransferase (EC 2.3.1.9) |
|  | CP095073.1_1756296_1757471 | 3-Ketoacyl-CoA thiolase [fadN-fadA-fadE operon] (EC 2.3.1.16) |
|  | CP095073.1_1753918_1756272 | 3-Hydroxyacyl-CoA dehydrogenase [fadN-fadA-fadE operon] (EC 1.1.1.35) / Enoyl-CoA hydratase [fadN-fadA-fadE operon] (EC 4.2.1.17) |
|  | CP095073.1_1966464_1967282 | Enoyl-CoA hydratase (EC 4.2.1.17) |
|  | CP095073.1_2827613_2826846 | Enoyl-CoA hydratase (EC 4.2.1.17) |
|  | CP095073.1_3209057_3208281 | Enoyl-CoA hydratase (EC 4.2.1.17) |
|  | CP095073.1_3589433_3588645 | Enoyl-CoA hydratase (EC 4.2.1.17) |
|  | CP095073.1_662202_663665 | Long-chain-fatty-acid--CoA ligase (EC 6.2.1.3) |
|  | CP095073.1_708127_709683 | Long-chain-fatty-acid--CoA ligase (EC 6.2.1.3) |
|  | CP095073.1_1242219_1243718 | Long-chain-fatty-acid--CoA ligase (EC 6.2.1.3) |
|  | CP095073.1_1964092_1965795 | Long-chain-fatty-acid--CoA ligase (EC 6.2.1.3) |
|  | CP095073.1_2597611_2595971 | Long-chain-fatty-acid--CoA ligase (EC 6.2.1.3) |
|  | CP095073.1_3174519_3172978 | Long-chain-fatty-acid--CoA ligase (EC 6.2.1.3) |
|  | CP095073.1_665644_666411 | 3-Oxoacyl-[acyl-carrier protein] reductase (EC 1.1.1.100) |
|  | CP095073.1_2753501_2752761 | 3-Oxoacyl-[acyl-carrier protein] reductase (EC 1.1.1.100), FadG |
|  | CP095073.1_3503574_3504344 | 3-Oxoacyl-[acyl-carrier protein] reductase (EC 1.1.1.100) |
| Protein folding | CP095073.1_2622153_2621449 | Cytochrome c-type biogenesis protein CcdA (DsbD analog) |
|  | CP095073.1_2104552_2105676 | Chaperone protein DnaJ |
|  | CP095073.1_2102586_2104412 | Chaperone protein DnaK |
|  | CP095073.1_2101993_2102544 | Heat shock protein GrpE |
|  | CP095073.1_2301928_2302362 | Peptidyl-prolyl cis-trans isomerase (EC 5.2.1.8) |
| Protein processing and modification | CP095073.1_188241_187744 | SSU ribosomal protein S5p (S2e) |
|  | CP095073.1_2467244_2467804 | Ribosomal-protein-S5p-alanine acetyltransferase (EC 2.3.1.128) |
|  | CP095073.1_4148784_4149335 | Ribosomal-protein-S5p-alanine acetyltransferase |
|  | CP095073.1_2797104_2796631 | Lipoprotein signal peptidase (EC 3.4.23.36) |
|  | CP095073.1_130697_131281 | Signal peptidase SipW (EC 3.4.21.89), required for TasA secretion |
|  | CP095073.1_2741853_2741305 | Signal peptidase I (EC 3.4.21.89) |
|  | CP095073.1_2919502_2920023 | Signal peptidase I (EC 3.4.21.89) |
|  | CP095073.1_3170057_3169512 | Signal peptidase I (EC 3.4.21.89) |
|  | CP095073.1_4049103_4048561 | Signal peptidase SipW (EC 3.4.21.89), required for TasA secretion |
|  | CP095073.1_1047026_1048186 | Glycosyl transferase, group 1 / Lipid carrier : UDP-N-acetylgalactosaminyltransferase (EC 2.4.1.-) |
| Cation transporter | CP095073.1_116335_114230 | Lead, cadmium, zinc and mercury transporting ATPase (EC 3.6.3.3) (EC 3.6.3.5); Copper-translocating P-type ATPase (EC 3.6.3.4) |
|  | CP095073.1_1470320_1472707 | Lead, cadmium, zinc and mercury transporting ATPase (EC 3.6.3.3) (EC 3.6.3.5); Copper-translocating P-type ATPase (EC 3.6.3.4) |
|  | CP095073.1_3434742_3432700 | Lead, cadmium, zinc and mercury transporting ATPase (EC 3.6.3.3) (EC 3.6.3.5); Copper-translocating P-type ATPase (EC 3.6.3.4) |
|  | CP095073.1_3562025_3563950 | Lead, cadmium, zinc and mercury transporting ATPase (EC 3.6.3.3) (EC 3.6.3.5); Copper-translocating P-type ATPase (EC 3.6.3.4) |
|  | CP095073.1_1469988_1470305 | Repressor CsoR of the copZA operon |
|  | CP095073.1_1472733_1472939 | Copper(I) chaperone CopZ |
|  | CP095073.1_2939468_2939070 | Magnesium and cobalt transport protein CorA |
|  | CP095073.1_2940019_2939465 | Magnesium and cobalt transport protein CorA |
| Multi-subunit cation antiporter | CP095073.1_899573_901435 | Na(+)/H(+) antiporter |
|  | CP095073.1_2081993_2079645 | Na(+) H(+) antiporter subunit A |
|  | CP095073.1_2621382_2620948 | Na(+) H(+) antiporter subunit B |
|  | CP095073.1_2079645_2079307 | Na(+) H(+) antiporter subunit C |
|  | CP095073.1_2079314_2077830 | Na(+) H(+) antiporter subunit D |
|  | CP095073.1_2077822_2077346 | Na(+) H(+) antiporter subunit E |
|  | CP095073.1_2077346_2077035 | Na(+) H(+) antiporter subunit F |
|  | CP095073.1_2077054_2076674 | Na(+) H(+) antiporter subunit G |
| Trehalose biosynthesis | CP095073.1_808562_809971 | PTS system, trehalose-specific IIB component (EC 2.7.1.201) / PTS system, trehalose-specific IIC component |
|  | CP095073.1_809995_811680 | Trehalose-6-phosphate hydrolase (EC 3.2.1.93) |
|  | CP095073.1_811704_812417 | Trehalose operon transcriptional repressor |
| Trehalose uptake and utilizaation | CP095073.1_1268483_1269199 | Trehalose utilization protein ThuA |
| Polysaccharides | CP095073.1_624648_626564 | 1,4-Alpha-glucan (glycogen) branching enzyme, GH-13-type (EC 2.4.1.18) |
|  | CP095073.1_627732_628838 | Glycogen biosynthesis protein GlgD, glucose-1-phosphate adenylyltransferase family |
|  | CP095073.1_628937_630367 | Glycogen synthase, ADP-glucose transglucosylase (EC 2.4.1.21) |
|  | CP095073.1_630392_632833 | Glycogen phosphorylase (EC 2.4.1.1) |
| **SSHM10-5^T^** | | |
| **Category** | **Location** | **Function** |
| Osmotic stress | CP095075.1_2129481_2130953 | Betaine aldehyde dehydrogenase (EC 1.2.1.8) |
|  | CP095075.1_2153529_2152045 | Betaine aldehyde dehydrogenase (EC 1.2.1.8) |
|  | CP095075.1_2151530_2150190 | Choline dehydrogenase (EC 1.1.99.1) |
|  | CP095075.1_2151931_2151482 | Choline dehydrogenase (EC 1.1.99.1) |
|  | CP095075.1_652186_650660 | Glycine betaine ABC transport system, permease protein OpuAB / Glycine betaine ABC transport system, glycine betaine-binding protein OpuAC |
|  | CP095075.1_1307380_1306448 | Glycine betaine ABC transport system, glycine betaine-binding protein OpuAC |
|  | CP095075.1_1911964_1912893 | Glycine betaine ABC transport system, glycine betaine-binding protein OpuAC |
|  | CP095075.1_2154537_2155421 | Glycine betaine ABC transport system, glycine betaine-binding protein OpuAC |
|  | CP095075.1_3948494_3949393 | Glycine betaine ABC transport system, glycine betaine-binding protein OpuAC |
|  | CP095075.1_1053891_1055426 | Glycine betaine transporter OpuD |
|  | CP095075.1_1364268_1362745 | Glycine betaine transporter OpuD |
|  | CP095075.1_2126516_2124975 | Glycine betaine transporter OpuD |
|  | CP095075.1_2267656_2269176 | Glycine betaine transporter OpuD |
|  | CP095075.1_3451833_3452804 | L-proline glycine betaine ABC transport system permease protein ProV (TC 3.A.1.12.1) |
|  | CP095075.1_3871152_3871331 | Glycerol uptake facilitator protein |
|  | CP095075.1_3921152_3921982 | Glycerol uptake facilitator protein |
|  | CP095074.1_4129022_4129873 | Glycine betaine ABC transport system, permease protein OpuAB |
|  | CP095075.1_1308312_1307461 | Glycine betaine ABC transport system, permease protein OpuAB |
|  | CP095075.1_2970957_2971787 | Glycine betaine ABC transport system, permease protein OpuAB |
| Oxidative stress | CP095075.1_452356_452820 | Ferric uptake regulation protein FUR |
|  | CP095075.1_2275590_2275174 | Organic hydroperoxide resistance protein |
|  | CP095075.1_1444689_1444252 | Peroxide stress regulator PerR, FUR family |
|  | CP095075.1_344838_345452 | Superoxide dismutase [Mn] (EC 1.15.1.1) |
|  | CP095075.1_1821622_1822587 | Superoxide dismutase [Mn] (EC 1.15.1.1) |
|  | CP095075.1_592131_592631 | Superoxide dismutase [Cu-Zn] precursor (EC 1.15.1.1) |
|  | CP095075.1_4001891_4002694 | Superoxide dismutase [Cu-Zn] precursor (EC 1.15.1.1) |
| Bacterial haemoglobins | CP095075.1_1219920_1220321 | Hemoglobin-like protein HbO |
|  | CP095075.1_1561525_1559345 | Diguanylate cyclase/phosphodiesterase (GGDEF & EAL domains) with PAS/PAC sensor(s) |
|  | CP095075.1_2061286_2060516 | Diguanylate cyclase/phosphodiesterase (GGDEF & EAL domains) with PAS/PAC sensor(s) |
|  | CP095075.1_2343378_2341243 | Diguanylate cyclase/phosphodiesterase (GGDEF & EAL domains) with PAS/PAC sensor(s) |
|  | CP095075.1_2416923_2417645 | Diguanylate cyclase/phosphodiesterase (GGDEF & EAL domains) with PAS/PAC sensor(s) |
|  | CP095075.1_2826931_2825618 | Diguanylate cyclase/phosphodiesterase (GGDEF & EAL domains) with PAS/PAC sensor(s) |
|  | CP095075.1_3461242_3463359 | Diguanylate cyclase/phosphodiesterase (GGDEF & EAL domains) with PAS/PAC sensor(s) |
|  | CP095075.1_3850762_3851127 | Diguanylate cyclase/phosphodiesterase (GGDEF & EAL domains) with PAS/PAC sensor(s) |
| Carbon starvation | CP095075.1_1968015_1966576 | Carbon starvation protein A |
|  | CP095075.1_2280857_2279418 | Carbon starvation protein A |
|  | CP095075.1_3064049_3062313 | Carbon starvation protein A |
|  | CP095075.1_3820973_3821200 | Carbon storage regulator |
| Dimethylarginine metabolism | CP095075.1_2030942_2031934 | NG,NG-dimethylarginine dimethylaminohydrolase 1 (EC 3.5.3.18) |
|  | CP095075.1_1261971_1263164 | Ornithine aminotransferase (EC 2.6.1.13) |
| Hfl operon | CP095075.1_678217_676985 | Ribosome LSU-associated GTP-binding protein HflX |
|  | CP095075.1_699346_699122 | RNA-binding protein Hfq |
| SigmaB stress response regulation | CP095075.1_2221768_2221367 | Anti-sigma B factor RsbT |
|  | CP095075.1_2220275_2219946 | Anti-sigma B factor antagonist RsbV |
|  | CP095075.1_2219495_2218716 | RNA polymerase sigma factor SigB |
|  | CP095075.1_3067232_3068062 | RsbR, positive regulator of sigma-B |
|  | CP095075.1_2222127_2221771 | RsbS, negative regulator of sigma-B |
|  | CP095075.1_2221350_2220340 | Serine phosphatase RsbU, regulator of sigma subunit |
|  | CP095075.1_2219943_2219467 | Serine-protein kinase RsbW (EC 2.7.11.1) |
| Amino acids and derivatives | CP095075.1_431144_432478 | NAD-specific glutamate dehydrogenase (EC 1.4.1.2); NADP-specific glutamate dehydrogenase (EC 1.4.1.4) |
|  | CP095075.1_498745_500016 | NAD-specific glutamate dehydrogenase (EC 1.4.1.2) |
|  | CP095075.1_1658299_1657274 | NAD-specific glutamate dehydrogenase (EC 1.4.1.2); NADP-specific glutamate dehydrogenase (EC 1.4.1.4) |
|  | CP095075.1_142464_143285 | Glutamate racemase (EC 5.1.1.3) |
|  | CP095075.1_2885787_2890379 | Glutamate synthase [NADPH] large chain (EC 1.4.1.13) |
|  | CP095075.1_2890404_2891891 | Glutamate synthase [NADPH] small chain (EC 1.4.1.13) |
|  | CP095075.1_3481154_3485764 | Glutamate synthase [NADPH] large chain (EC 1.4.1.13) |
|  | CP095075.1_3485784_3487268 | Glutamate synthase [NADPH] small chain (EC 1.4.1.13) |
|  | CP095075.1_1043534_1042602 | Glutaminase (EC 3.5.1.2) |
|  | CP095075.1_2406106_2407050 | Glutaminase (EC 3.5.1.2) |
|  | CP095075.1_675176_673845 | Glutamine synthetase type I (EC 6.3.1.2) |
|  | CP095075.1_1575055_1576365 | Glutamine synthetase type I (EC 6.3.1.2) |
|  | CP095075.1_3555773_3557320 | Delta-1-pyrroline-5-carboxylate dehydrogenase (EC 1.2.1.88) |
|  | CP095075.1_2064932_2065051 | Proline/sodium symporter PutP (TC 2.A.21.2.1) @ Propionate/sodium symporter |
|  | CP095075.1_2065069_2065302 | Proline/sodium symporter PutP (TC 2.A.21.2.1) @ Propionate/sodium symporter |
|  | CP095075.1_2072614_2071103 | Proline/sodium symporter PutP (TC 2.A.21.2.1) @ Propionate/sodium symporter |
|  | CP095075.1_1600424_1599162 | Gamma-glutamyl phosphate reductase (EC 1.2.1.41) |
|  | CP095075.1_1601612_1600479 | Glutamate 5-kinase (EC 2.7.2.11) / RNA-binding C-terminal domain PUA |
|  | CP095075.1_1411217_1410402 | Pyrroline-5-carboxylate reductase (EC 1.5.1.2) |
|  | CP095075.1_1602512_1601670 | Pyrroline-5-carboxylate reductase (EC 1.5.1.2) |
| Iron acquisition and metabolism | CP095075.1_2446446_2445691 | Uncharacterized iron compound ABC uptake transporter, ATP-binding protein |
|  | CP095075.1_2447387_2446443 | Uncharacterized iron compound ABC uptake transporter, permease protein |
|  | CP095075.1_2448330_2447377 | Uncharacterized iron compound ABC uptake transporter, permease protein |
|  | CP095075.1_2445667_2444735 | Uncharacterized iron compound ABC uptake transporter, substrate-binding protein |
|  | CP095075.1_2760476_2761180 | Two-component response regulator SA14-24 |
|  | CP095075.1_2761187_2763025 | Two-component sensor kinase SA14-24 |
|  | CP095075.1_2765249_2766043 | Zn-dependent hydrolase YycJ/WalJ, required for cell wall metabolism and coordination of cell division with DNA replication |
| Potassium homeostasis | CP095075.1_2400604_2400122 | Large-conductance mechanosensitive channel |
|  | CP095075.1_1075514_1076359 | Potassium efflux system KefA protein / Small-conductance mechanosensitive channel |
|  | CP095075.1_1543128_1543988 | Potassium efflux system KefA protein / Small-conductance mechanosensitive channel |
|  | CP095075.1_2737749_2738633 | Potassium efflux system KefA protein / Small-conductance mechanosensitive channel |
|  | CP095075.1_3251734_3250649 | Potassium efflux system KefA protein / Small-conductance mechanosensitive channel |
|  | CP095075.1_4048942_4049967 | Potassium voltage-gated channel subfamily KQT; possible potassium channel, VIC family |
|  | CP095075.1_1448523_1448116 | Potassium channel protein |
|  | CP095075.1_3328932_3329150 | 3-Hydroxyacyl-[acyl-carrier-protein] dehydratase, FabZ form (EC 4.2.1.59) |
|  | CP095075.1_3329147_3329353 | 3-Hydroxyacyl-[acyl-carrier-protein] dehydratase, FabZ form (EC 4.2.1.59) |
|  | CP095075.1_4127596_4128456 | Acetyl-coenzyme A carboxyl transferase beta chain (EC 6.4.1.2) |
|  | CP095075.1_4128453_4129415 | Acetyl-coenzyme A carboxyl transferase alpha chain (EC 6.4.1.2) |
|  | CP095075.1_829468_829235 | Acyl carrier protein |
|  | CP095075.1_390023_390520 | Biotin carboxyl carrier protein of acetyl-CoA carboxylase |
|  | CP095075.1_390536_391405 | Biotin carboxylase of acetyl-CoA carboxylase (EC 6.3.4.14) |
|  | CP095075.1_391402_391908 | Biotin carboxylase of acetyl-CoA carboxylase (EC 6.3.4.14) |
|  | CP095075.1_641637_640282 | Biotin carboxylase of acetyl-CoA carboxylase (EC 6.3.4.14) |
|  | CP095075.1_1109727_1108948 | Enoyl-[acyl-carrier-protein] reductase [NADH] (EC 1.3.1.9) |
|  | CP095075.1_1210527_1209751 | Enoyl-[acyl-carrier-protein] reductase [NADH] (EC 1.3.1.9) |
|  | CP095075.1_831209_830268 | Malonyl CoA-acyl carrier protein transacylase (EC 2.3.1.39) |
|  | CP095075.1_1728454_1727525 | Malonyl CoA-acyl carrier protein transacylase (EC 2.3.1.39) |
|  | CP095075.1_2860394_2861350 | 3-Ketoacyl-CoA thiolase (EC 2.3.1.16) @ Acetyl-CoA acetyltransferase (EC 2.3.1.9) |
|  | CP095075.1_2922190_2923344 | 3-Ketoacyl-CoA thiolase (EC 2.3.1.16) @ Acetyl-CoA acetyltransferase (EC 2.3.1.9) |
|  | CP095075.1_3566979_3568148 | 3-Ketoacyl-CoA thiolase (EC 2.3.1.16) @ Acetyl-CoA acetyltransferase (EC 2.3.1.9) |
|  | CP095075.1_3602081_3603265 | 3-Ketoacyl-CoA thiolase (EC 2.3.1.16) @ Acetyl-CoA acetyltransferase (EC 2.3.1.9) |
|  | CP095075.1_3959421_3960596 | 3-Ketoacyl-CoA thiolase [fadN-fadA-fadE operon] (EC 2.3.1.16) |
|  | CP095075.1_21482_22255 | Enoyl-CoA hydratase (EC 4.2.1.17) |
|  | CP095075.1_1016841_1015981 | Enoyl-CoA hydratase (EC 4.2.1.17) |
|  | CP095075.1_1578235_1577447 | Enoyl-CoA hydratase (EC 4.2.1.17) |
|  | CP095075.1_3400546_3401352 | Enoyl-CoA hydratase (EC 4.2.1.17) |
|  | CP095075.1_3603517_3604296 | Enoyl-CoA hydratase (EC 4.2.1.17) |
|  | CP095075.1_3957000_3959399 | 3-Hydroxyacyl-CoA dehydrogenase [fadN-fadA-fadE operon] (EC 1.1.1.35) / Enoyl-CoA hydratase [fadN-fadA-fadE operon] (EC 4.2.1.17) |
|  | CP095075.1_19079_20788 | Long-chain-fatty-acid--CoA ligase (EC 6.2.1.3) |
|  | CP095075.1_643293_641653 | Long-chain-fatty-acid--CoA ligase (EC 6.2.1.3) |
|  | CP095075.1_1288728_1287196 | Long-chain-fatty-acid--CoA ligase (EC 6.2.1.3) |
|  | CP095075.1_2025941_2024355 | Long-chain-fatty-acid--CoA ligase (EC 6.2.1.3) |
|  | CP095075.1_2134683_2133073 | Long-chain-fatty-acid--CoA ligase (EC 6.2.1.3) |
|  | CP095075.1_2492303_2493955 | Long-chain-fatty-acid--CoA ligase (EC 6.2.1.3) |
|  | CP095075.1_2858745_2860214 | Long-chain-fatty-acid--CoA ligase (EC 6.2.1.3) |
|  | CP095075.1_2924240_2925796 | Long-chain-fatty-acid--CoA ligase (EC 6.2.1.3) |
|  | CP095075.1_3570400_3572079 | Long-chain-fatty-acid--CoA ligase (EC 6.2.1.3) |
|  | CP095075.1_3595349_3596974 | Long-chain-fatty-acid--CoA ligase (EC 6.2.1.3) |
|  | CP095075.1_722467_721748 | 3-Oxoacyl-[acyl-carrier protein] reductase (EC 1.1.1.100) |
|  | CP095075.1_830268_829528 | 3-Oxoacyl-[acyl-carrier protein] reductase (EC 1.1.1.100), FadG |
| Protein folding | CP095075.1_65070_65780 | Cytochrome c-type biogenesis protein CcdA (DsbD analog) |
|  | CP095075.1_667966_667262 | Cytochrome c-type biogenesis protein CcdA (DsbD analog) |
|  | CP095075.1_308050_309171 | Chaperone protein DnaJ |
|  | CP095075.1_306078_307907 | Chaperone protein DnaK |
|  | CP095075.1_305493_306041 | Heat shock protein GrpE |
|  | CP095075.1_467160_467600 | Peptidyl-prolyl cis-trans isomerase (EC 5.2.1.8) |
|  | CP095075.1_468016_468777 | Peptidyl-prolyl cis-trans isomerase (EC 5.2.1.8) |
| Protein processing and modification | CP095075.1_2535094_2534597 | SSU ribosomal protein S5p (S2e) |
|  | CP095075.1_738166_737624 | Ribosomal-protein-S5p-alanine acetyltransferase (EC 2.3.1.128) |
|  | CP095075.1_978604_978131 | Lipoprotein signal peptidase (EC 3.4.23.36) |
|  | CP095075.1_813770_813222 | Signal peptidase I (EC 3.4.21.89) |
|  | CP095075.1_1089866_1090384 | Signal peptidase I (EC 3.4.21.89) |
|  | CP095075.1_1284256_1283717 | Signal peptidase I (EC 3.4.21.89) |
|  | CP095075.1_1998691_1998092 | Lipid carrier : UDP-N-acetylgalactosaminyltransferase (EC 2.4.1.-) |
| Cation transporter | CP095075.1_69043_71127 | Lead, cadmium, zinc and mercury transporting ATPase (EC 3.6.3.3) (EC 3.6.3.5); Copper-translocating P-type ATPase (EC 3.6.3.4) |
|  | CP095075.1_75262_77667 | Lead, cadmium, zinc and mercury transporting ATPase (EC 3.6.3.3) (EC 3.6.3.5); Copper-translocating P-type ATPase (EC 3.6.3.4) |
|  | CP095075.1_1553682_1555604 | Lead, cadmium, zinc and mercury transporting ATPase (EC 3.6.3.3) (EC 3.6.3.5); Copper-translocating P-type ATPase (EC 3.6.3.4) |
|  | CP095075.1_3187584_3188624 | Lead, cadmium, zinc and mercury transporting ATPase (EC 3.6.3.3) (EC 3.6.3.5); Copper-translocating P-type ATPase (EC 3.6.3.4) |
|  | CP095075.1_3188621_3189040 | Lead, cadmium, zinc and mercury transporting ATPase (EC 3.6.3.3) (EC 3.6.3.5); Copper-translocating P-type ATPase (EC 3.6.3.4) |
|  | CP095075.1_3189088_3189987 | Lead, cadmium, zinc and mercury transporting ATPase (EC 3.6.3.3) (EC 3.6.3.5); Copper-translocating P-type ATPase (EC 3.6.3.4) |
|  | CP095075.1_3191123_3193663 | Lead, cadmium, zinc and mercury transporting ATPase (EC 3.6.3.3) (EC 3.6.3.5); Copper-translocating P-type ATPase (EC 3.6.3.4) |
|  | CP095075.1_74929_75246 | Repressor CsoR of the copZA operon |
|  | CP095075.1_3187253_3187570 | Repressor CsoR of the copZA operon |
|  | CP095075.1_77691_77894 | Copper(I) chaperone CopZ |
|  | CP095075.1_3190013_3190216 | Copper(I) chaperone CopZ |
|  | CP095075.1_1104542_1103589 | Magnesium and cobalt transport protein CorA |
| Multi-subunit cation antiporter | CP095075.1_3171929_3173800 | Na(+)/H(+) antiporter |
|  | CP095075.1_286120_283784 | Na(+) H(+) antiporter subunit A |
|  | CP095075.1_667194_666766 | Na(+) H(+) antiporter subunit B |
|  | CP095075.1_283780_283442 | Na(+) H(+) antiporter subunit C |
|  | CP095075.1_283449_281965 | Na(+) H(+) antiporter subunit D |
|  | CP095075.1_281957_281481 | Na(+) H(+) antiporter subunit E |
|  | CP095075.1_281481_281170 | Na(+) H(+) antiporter subunit F |
|  | CP095075.1_281189_280818 | Na(+) H(+) antiporter subunit G |
| Trehalose biosynthesis | CP095075.1_3088306_3089991 | Trehalose-6-phosphate hydrolase (EC 3.2.1.93) |
|  | CP095075.1_3090016_3090729 | Trehalose operon transcriptional repressor |
|  | CP095075.1_3086867_3088276 | PTS system, trehalose-specific IIB component (EC 2.7.1.201) / PTS system, trehalose-specific IIC component |
| Trehalose uptake and utilizaation | CP095075.1_3715075_3715794 | Trehalose utilization protein ThuA |
|  | CP095075.1_1780502_1779831 | Beta-phosphoglucomutase (EC 5.4.2.6) |
| Polysaccharides | CP095075.1_2847028_2848947 | 1,4-Alpha-glucan (glycogen) branching enzyme, GH-13-type (EC 2.4.1.18) |
|  | CP095075.1_2849824_2850096 | Glucose-1-phosphate adenylyltransferase (EC 2.7.7.27) |
|  | CP095075.1_2850110_2850469 | Glycogen biosynthesis protein GlgD, glucose-1-phosphate adenylyltransferase family |
|  | CP095075.1_2848937_2849827 | Glucose-1-phosphate adenylyltransferase (EC 2.7.7.27) |
|  | CP095075.1_2849824_2850096 | Glucose-1-phosphate adenylyltransferase (EC 2.7.7.27) |
|  | CP095075.1_2851505_2852935 | Glycogen synthase, ADP-glucose transglucosylase (EC 2.4.1.21) |
|  | CP095075.1_2852960_2855398 | Glycogen phosphorylase (EC 2.4.1.1) |

**Supplementary Table S4** : List of CYP oxidase genes identified in *Halobacillus* strains SSTM10-2^T^, SSBR10-3^T^, and SSHM10-5^T^.

| ***Halalobacillus shinanisalinarum* SSTM10-2^T^** | ***Halobacillus salinarum* SSBR10-3^T^** | ***Halobacillus amylolyticus* SSHM10-5^T^** |
| --- | --- | --- |
| Cytochrome c oxidase caa3-type assembly factor CtaG_BS (unrelated to Cox11-CtaG family) | Cytochrome d ubiquinol oxidase subunit I (EC 1.10.3.-) | Cytochrome c oxidase polypeptide III (EC 1.9.3.1) |
| Cytochrome c oxidase caa3-type assembly factor CtaG_BS (unrelated to Cox11-CtaG family) | Cytochrome d ubiquinol oxidase subunit II (EC 1.10.3.-) | Cytochrome c oxidase polypeptide I (EC 1.9.3.1) |
| Heme A synthase, cytochrome oxidase biogenesis protein Cox15-CtaA | Cytochrome d ubiquinol oxidase subunit I (EC 1.10.3.-) | Cytochrome c oxidase polypeptide II (EC 1.9.3.1) |
| Cytochrome c oxidase polypeptide I (EC 1.9.3.1) | Cytochrome d ubiquinol oxidase subunit II (EC 1.10.3.-) | Cytochrome d ubiquinol oxidase subunit II (EC 1.10.3.-) |
| Cytochrome c oxidase polypeptide II (EC 1.9.3.1) | Cytochrome c oxidase polypeptide II (EC 1.9.3.1) | Cytochrome d ubiquinol oxidase subunit I (EC 1.10.3.-) |
| Cytochrome aa3-600 menaquinol oxidase subunit II | Cytochrome c oxidase polypeptide I (EC 1.9.3.1) | Cytochrome c oxidase polypeptide II (EC 1.9.3.1) |
| Cytochrome aa3-600 menaquinol oxidase subunit I | Cytochrome c oxidase polypeptide III (EC 1.9.3.1) | Cytochrome c oxidase polypeptide I (EC 1.9.3.1) |
| Cytochrome aa3-600 menaquinol oxidase subunit III | Heme A synthase, cytochrome oxidase biogenesis protein Cox15-CtaA | Cytochrome c oxidase caa3-type assembly factor CtaG_BS (unrelated to Cox11-CtaG family) |
| Cytochrome aa3-600 menaquinol oxidase subunit IV | Cytochrome aa3-600 menaquinol oxidase subunit II | Cytochrome c oxidase caa3-type assembly factor CtaG_BS (unrelated to Cox11-CtaG family) |
| Cytochrome oxidase biogenesis protein Sco1/SenC/PrrC, thiol-disulfide reductase involved in Cu(I) insertion into CoxII Cu(A) center | Cytochrome aa3-600 menaquinol oxidase subunit I | Cytochrome c oxidase polypeptide IV (EC 1.9.3.1) |
| Cytochrome oxidase biogenesis protein Sco1/SenC/PrrC, thiol-disulfide reductase involved in Cu(I) insertion into CoxII Cu(A) center | Cytochrome aa3-600 menaquinol oxidase subunit I | Cytochrome oxidase biogenesis protein Sco1/SenC/PrrC, thiol-disulfide reductase involved in Cu(I) insertion into CoxII Cu(A) center |
| Cytochrome d ubiquinol oxidase subunit I (EC 1.10.3.-) | Cytochrome aa3-600 menaquinol oxidase subunit III | Cytochrome c oxidase caa3-type assembly factor CtaG_BS (unrelated to Cox11-CtaG family) |
| Cytochrome d ubiquinol oxidase subunit II (EC 1.10.3.-) | AA3-600 quinol oxidase subunit IV | Cytochrome oxidase biogenesis protein Sco1/SenC/PrrC, thiol-disulfide reductase involved in Cu(I) insertion into CoxII Cu(A) center |
| Cytochrome c oxidase polypeptide II (EC 1.9.3.1) | Cytochrome c oxidase caa3-type assembly factor CtaG_BS (unrelated to Cox11-CtaG family) | Cytochrome aa3-600 menaquinol oxidase subunit IV |
| Cytochrome c oxidase polypeptide I (EC 1.9.3.1) | Cytochrome c oxidase polypeptide IV (EC 1.9.3.1) | Cytochrome aa3-600 menaquinol oxidase subunit III |
| Cytochrome c oxidase polypeptide III (EC 1.9.3.1) | Cytochrome c oxidase polypeptide III (EC 1.9.3.1) | Cytochrome aa3-600 menaquinol oxidase subunit I |
| Cytochrome c oxidase polypeptide IV (EC 1.9.3.1) | Cytochrome c oxidase polypeptide I (EC 1.9.3.1) | Cytochrome aa3-600 menaquinol oxidase subunit II |
| Cytochrome c oxidase caa3-type assembly factor CtaG_BS (unrelated to Cox11-CtaG family) | Cytochrome c oxidase polypeptide II (EC 1.9.3.1) |  |
|  | Cytochrome d ubiquinol oxidase subunit II (EC 1.10.3.-) |  |
|  | Cytochrome d ubiquinol oxidase subunit I (EC 1.10.3.-) |  |
|  | Cytochrome oxidase biogenesis protein Sco1/SenC/PrrC, thiol-disulfide reductase involved in Cu(I) insertion into CoxII Cu(A) center |  |
|  | Cytochrome oxidase biogenesis protein Sco1/SenC/PrrC, thiol-disulfide reductase involved in Cu(I) insertion into CoxII Cu(A) center |  |
|  | Cytochrome oxidase biogenesis protein Sco1/SenC/PrrC, thiol-disulfide reductase involved in Cu(I) insertion into CoxII Cu(A) center |  |
